# Promising New Inhibitors of Tyrosyl-DNA Phosphodiesterase I (Tdp 1) Combining 4-Arylcoumarin and Monoterpenoid Moieties as Components of Complex Antitumor Therapy

**DOI:** 10.3390/ijms21010126

**Published:** 2019-12-23

**Authors:** Tatyana M. Khomenko, Alexandra L. Zakharenko, Arina A. Chepanova, Ekaterina S. Ilina, Olga D. Zakharova, Vasily I. Kaledin, Valeriy P. Nikolin, Nelly A. Popova, Dina V. Korchagina, Jóhannes Reynisson, Raina Chand, Daniel M. Ayine-Tora, Jinal Patel, Ivanhoe K. H. Leung, Konstantin P. Volcho, Nariman F. Salakhutdinov, Olga I. Lavrik

**Affiliations:** 1 N.N. Vorozhtsov Novosibirsk Institute of Organic Chemistry, 9 acad. Lavrentjev ave., 630090 Novosibirsk, Russia; chomenko@nioch.nsc.ru (T.M.K.); korchaga@nioch.nsc.ru (D.V.K.); anvar@nioch.nsc.ru (N.F.S.); 2 Novosibirsk Institute of Chemical Biology and Fundamental Medicine, 8, acad. Lavrentjev ave., 630090 Novosibirsk, Russia; sashaz@niboch.nsc.ru (A.L.Z.); arinachepanova@mail.ru (A.A.C.); katya.plekhanova@gmail.com (E.S.I.); garonna3@mail.ru (O.D.Z.); lavrik@niboch.nsc.ru (O.I.L.); 3 Institute of Cytology and Genetics, 10, acad. Lavrentjev Ave., 630090 Novosibirsk, Russian; kaledin@bionet.nsc.ru (V.I.K.); nikolin@bionet.nsc.ru (V.P.N.); nelly@bionet.nsc.ru (N.A.P.); 4 Novosibirsk State University, V. Zelman Institute for Medicine and Psychology and Department of Natural Sciences, 2, Pirogova str., 630090 Novosibirsk, Russia; 5 School of Pharmacy and Bioengineering, Keele University, Hornbeam Building, Staffordshire ST5 5BG, UK; j.reynisson@keele.ac.uk; 6 School of Chemical Sciences, The University of Auckland, Private Bag 92019, 1142 Auckland, New Zealand; rcha387@aucklanduni.ac.nz (R.C.); dayi479@aucklanduni.ac.nz (D.M.A.-T.); jpat649@aucklanduni.ac.nz (J.P.); i.leung@auckland.ac.nz (I.K.H.L.); 7 Department of Physical and Chemical Biology and Biotechnology, Altai State University, 61, Lenina Ave., 656049 Barnaul, Russia

**Keywords:** coumarin, neoflavone, DNA repair enzymes, Tdp1 inhibitor, cancer, tumor, topotecan, topoisomerase 1 inhibitors, molecular modeling, chemical space

## Abstract

Tyrosyl-DNA phosphodiesterase 1 (Tdp1) is an important DNA repair enzyme in humans, and a current and promising inhibition target for the development of new chemosensitizing agents due to its ability to remove DNA damage caused by topoisomerase 1 (Top1) poisons such as topotecan and irinotecan. Herein, we report our work on the synthesis and characterization of new Tdp1 inhibitors that combine the arylcoumarin (neoflavonoid) and monoterpenoid moieties. Our results showed that they are potent Tdp1 inhibitors with IC_50_ values in the submicromolar range. In vivo experiments with mice revealed that compound **3ba** (IC_50_ 0.62 µM) induced a significant increase in the antitumor effect of topotecan on the Krebs-2 ascites tumor model. Our results further strengthen the argument that Tdp1 is a druggable target with the potential to be developed into a clinically-potent adjunct therapy in conjunction with Top1 poisons.

## 1. Introduction

**Figure 1:**
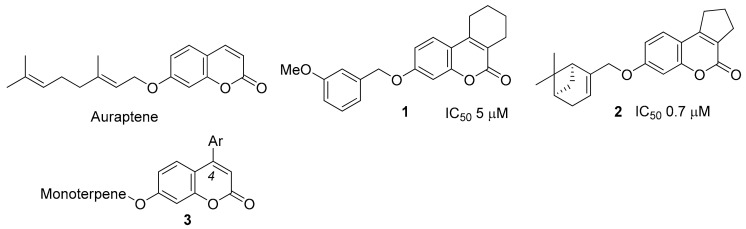
Examples of biologically-active coumarins: auraptene and compounds **1**–**3**.

Natural and synthetic coumarins (2*H*-chromen-2-one) demonstrate diverse biological activities, and are often considered as a privileged scaffold [1,2,3,4,5,6]. In particular, a large number of coumarin derivatives with high antitumor activity have been found in recent years [7,8,9,10,11,12,13,14,15,16,17,18]. Natural derivatives of 7-hydroxycoumarin containing terpene fragments have also attracted the attention of the medicinal chemistry community [19,20,21]. The best-known compound of this structural type is auraptene (Figure 1), for which a variety of biological activities are known, including antitumor properties [22].

One of the current approaches to increase the efficacy of clinically-established antitumor therapy is the inhibition of DNA repair enzymes that counteract the effect of DNA-damaging chemotherapy agents [23,24,25]. One of these important enzymes is tyrosyl-DNA phosphodiesterase 1 (Tdp1) [26]. Tdp1 is involved in the repairing of damaged DNA, including the removal of lesions caused by topoisomerase 1 (Top1) inhibitors. Top1 inhibitors such as the camptothecin derivatives (CPTs), topotecan (tpc), and irinotecan are well-established antitumor agents [27] that are widely used [28]. Thus, Tdp1 reduces the impact of Top1 poisons, resulting in diminished DNA damage and reduced efficacy of this class of chemotherapeutic drugs.

Because Tdp1 repairs Top1/DNA cleavage complexes induced by CPTs, inhibitors of Tdp1 can enhance the sensitivity of cancer cells to CPT analogues [29]. Furthermore, increased Tdp1 expression counteracts the cytotoxicity of CPTs [30,31], and is frequently observed in cancers resistant to CPT therapy [31,32,33]. Convincing evidence exists from preclinical studies that the ratio of Tdp1/Top1 activity influences cellular sensitivity to Top1 inhibitors [34,35], and that the suppression of Tdp1 activity leads to an increase in the sensitivity of tumor cells to CPTs [32,36,37,38,39]. It is believed that targeted short-term treatment with a potent Tdp1 inhibitor will not lead to serious poisoning in normal cells. Indeed, it was shown that Tdp1-/- knockout mice were fertile and had a normal life expectancy, with no signs of premature aging [40]. Until now, no inhibitors of the Tdp1 enzyme have reached human clinical testing. 

To date, many Tdp1 inhibitors have been identified. A major class of Tdp1 inhibitors comprises those based on natural products including usnic acid derivatives [41,42,43,44,45], coumarins [46], adamantanes [47,48,49], nucleoside analogs [50], dehydroabietylamine derivatives [51], chromenes [52], bile acids derivatives [53], and fungal products [54,55,56]. There are also early reports of Tdp1 inhibition based on diamidines [57], antibiotics [58,59], steroids [60], and other compounds [61]. Nevertheless, only a few Tdp1 inhibitors have been tested in cell- or cancer-models. Synergy with tpc has been demonstrated in vivo for just two Tdp1 inhibitors, both of which were derivatives of usnic acid, a natural product [43,44]. 

Previously, virtual screening of the InterBioScreen natural product library [62] and subsequent testing identified that 3-methoxybenzyl, a derivative of 7-hydroxycoumarin, annelated with the cyclohexane ring **1** (Figure 1) as a new structural type of Tdp1 inhibitors [46]. Further optimization of the inhibitor, including the replacement of the aromatic substituent in the phenolic group with bulky monoterpenoid substituents, allowed us to increase the potency by almost an order of magnitude, thereby reaching nanomolar activity [46]. Most importantly, the use of compound **2** in non-toxic doses significantly increased the cytotoxic activity of CPT in human cancer cells [46].

Based on molecular modeling, it was predicted that the attachment of an aromatic substituent at the fourth position of coumarin would be promising for enhanced binding. Note that 4-arylcoumarins are often considered a separate group of natural products, called neoflavones. Natural and synthetic neoflavones have low toxicities and exhibit a broad spectrum of biological activity, in particular against tumors [7]. The aim of this work was to synthesize neoflavone derivatives of structural type 3 (Figure 1) by varying both aromatic and monoterpene substituents to determine their inhibitory activity against Tdp1, and to study the synergistic effect with tpc, a clinically-important Top1 inhibitor, in in vivo experiments. As a result, it was discovered that arylcoumarins containing monoterpenoid substituents are indeed potent Tdp1 inhibitors and, most importantly, are able to enhance the antitumor activity of tpc in animal models.

## 2. Results and Discussion

### 2.1. Chemistry 

**Scheme 1:**
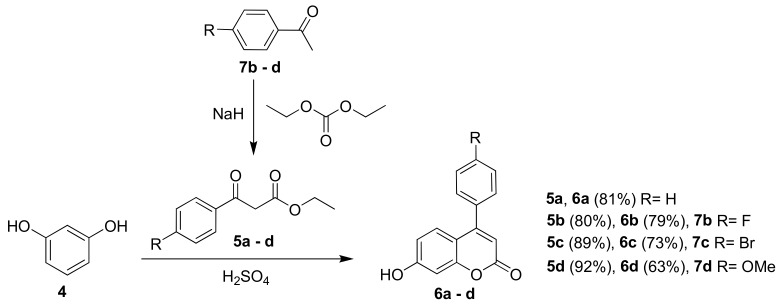
Synthesis of 7-hydroxy-4-arylcoumarins **6a–d.**

The main approach to producing 4-arylcoumarins unsubstituted at the hydroxy group is acid-catalyzed Pechmann condensation between resorcinol **4** and ester of β-keto-carboxylic acids **5** [63] (Scheme 1). Using this approach, we synthesized 7-hydroxy-4-arylcoumarins **6a–d** with yields of 63-81% by interaction of resorcinol **4** with esters **5a–d** (Scheme 1). Ester **5a** is commercially available, while compounds **5b–d** were obtained by the reaction of substituted acetophenones **5b–d** with diethyl carbonate in the presence of sodium hydride.

**Scheme 2:**
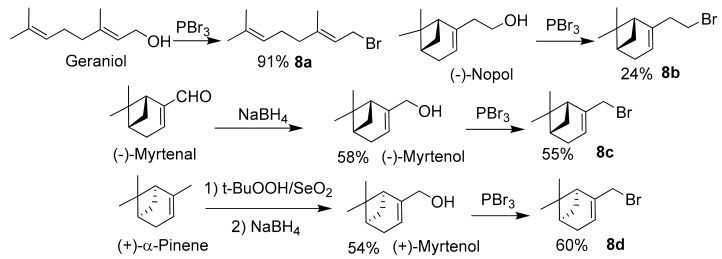
Synthesis of monoterpenoid bromides **8a–d**.

Monoterpenoid bromides **8a–d** were obtained from the corresponding alcohols (geraniol, (−)-nopol, (−)- and (+)-myrtenols) by interaction with PBr_3_ according to the procedure [46] (Scheme 2). Geraniol and (−)-nopol were purchased from commercial sources, while (−)- and (+)-myrtenols were synthesized from (−)-myrtenal and (+)-α-pinene in accordance with the methods [46]. The choice of monoterpenoids was based on the results we obtained previously, i.e., when both the absolute configuration of the pinane cycle and the length of the bridge played important roles [46]. A further consideration was the desire to compare the activity data obtained with bulky bicyclic substituents with corresponding data for products with acyclic monoterpene fragments. Note that coumarin-containing derivatives of geraniol can be considered as analogues of the natural coumarin auraptene, having the same monoterpenoid fragment (Figure 1).

**Scheme 3:**
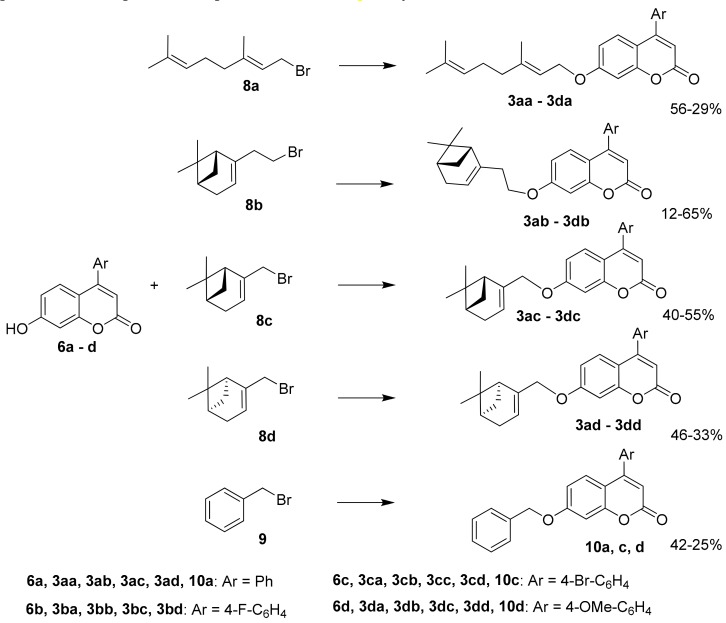
Synthesis of monoterpenoid-arylcoumarin hybrids.

The target monoterpenoid-arylcoumarin hybrids **3** were synthesized by the reaction of 7-hydroxycoumarins **6a–d** with monoterpenoid bromides **8a–d** using DBU in DMF (Scheme 3). To compare and identify the importance of the monoterpenoid fragment, compounds **10a, c, d** containing a benzyl substituent were also synthesized. The products were purified by recrystallization or column chromatography, and obtained with yields of 12–65%. In the case of the synthesis of nopol derivatives, a low conversion was observed, and double purification on SiO_2_ was required, for example, for compounds **3ab** or **3cb** with yields less than 20%).

### 2.2. Biology

A previously designed [64], real-time, hexadecameric oligonucleotide biosensor with 5(6)-carboxyfluorescein (FAM) at the 5′ end and fluorophore quencher BHQ1 (Black Hole Quencher-1) at the 3′-end was used to determine the inhibitory properties of the new compounds.

**Figure 2:**
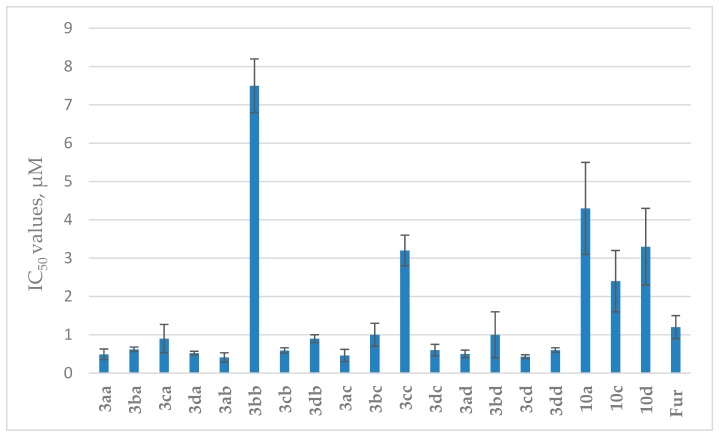
The Tdp1 inhibitory activities of compounds **3aa**–**3dd** and **10a**, **c**, **d**. Furamidine (Fur) was used as a positive control.

The results of the Tdp1 assay for the arylcoumarin derivatives are shown in Figure 2 and Supplementary Table S1. All arylcoumarin derivatives containing a geraniol residue (**3aa–3da**) showed high inhibitory activity, with IC_50_ values in the submicromolar range; compound **3ac** with a bromine atom in the aromatic ring was slightly less active. Among the derivatives of nopol **3ab–3db**, only the fluorine derivative **3bb** showed a markedly lower activity; the remaining compounds were comparable in activity with the derivatives of geraniol. Since it was necessary to use column chromatography to obtain nopol-arylcoumarin hybrids, which complicates and increases the cost of the synthesis process, geraniol-containing inhibitors are more promising for further studies.

Almost all derivatives of (−)- and (+)-myrtenols (**3ac–3dc** and **3ad–3dd**, respectively) showed similar inhibitory activity with IC_50_ values in the 0.4-1.0 µM range, except compound **3cc**. Interestingly, compounds **10a, c, d** containing a benzyl substituent instead of monoterpenoid fragments were significantly less active than most of their monoterpenoid-containing analogues, with an IC_50_ in the micromolar range.

**Figure 3:**
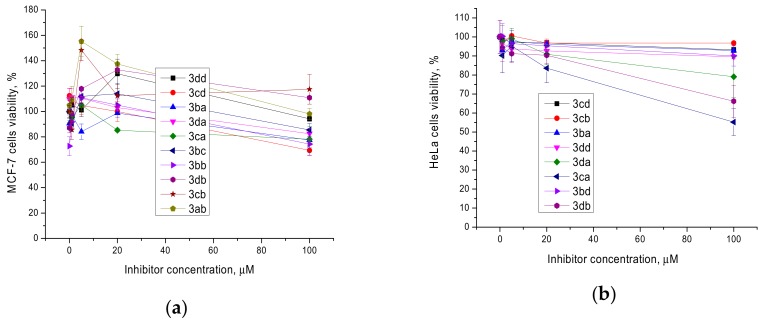
The effect of compounds **3** on the survival of cells of the lines MCF-7 (**a**) and HeLa (**b**).

An analysis of the cytotoxicity of the synthesized compounds was performed on cell lines of human breast adenocarcinoma MCF-7 and human cervical cancer HeLa. It turned out that cytotoxicity is absent or insignificant in the entire range of studied concentrations (up to 100 μM) for all the tested compounds, which makes it possible to use them as tumor sensitizers for currently-used antitumor drugs without introducing additional toxic burden (Figure 3).

Since most monoterpene-arylcoumarin hybrids showed comparable inhibitory activity against Tdp1 (~0.5 μM) and no or limited cytotoxicity, we selected a candidate for subsequent studies based on the following considerations. Derivatives of nopol were the most complex compounds to synthesize and purify. Therefore, they were excluded from further consideration. Since we previously obtained contradictory results in in vivo experiments with the myrtenol derivative **2** (unpublished data), in this work, we decided to focus on geraniol derivatives for the in vivo studies. Among the three derivatives of geraniol that showed similar inhibitory activity (Figure 2), we selected compound **3ba** containing a fluorine atom in the para position of the aryl substituent, which can contribute to greater metabolic stability of the inhibitor [65]. In addition to the activity assay that we reported above, we wanted to confirm the interactions between compound **3ba** and Tdp1 before progressing to in vivo studies. Using an intrinsic tryptophan fluorescence quenching assay that we previously applied to study the binding interactions of Tdp1 and its inhibitors [43,45,48,49,52], we evaluated the binding of compound **3ba** to recombinant Tdp1. Clear quenching of the Tdp1 intrinsic fluorescence was observed upon the addition of **3ba** (Supplementary Figure S1). Titration experiments were then performed to determine the dissociation constant (*K*_D_) of compound **3ba** with Tdp1. A *K*_D_ value of 63.0 ± 11 μM was obtained, indicating that it is a reasonable binder to the enzyme. This confirmed that the inhibition efficacy of compound **3ba** was due to binding to the enzyme, and gave us confidence to progress with this compound towards in vivo studies.

A study of the influence of **3ba** on the antitumor effect of tpc (topotecan) was performed using a murine Krebs-2 carcinoma model. An ascitic tumor model combines the advantages of in vitro and in vivo approaches in studying the cytotoxic effect of compounds, since ascitic cells grow in the context of the organism (in vivo), and the intraperitoneal administration of drug ensures its direct contact with tumor cells (in vitro). The experiments were performed using female C57BL/6 mice, which were injected intraperitoneally with 2 × 10^5^ ascitic cells on day zero. The mice were divided into six groups of ten animals each. Control group 1 did not receive treatment; group 2 received tpc in a single dose of 0.5 mg/kg of body weight intraperitoneally after 2 days; group 3 received tpc as described above, and **3ba** at a dose of 80 mg/kg intraperitoneally; group 4 received tpc and **3ba** 40 mg/kg; group 5 received tpc and **3ba** 20 mg/kg; and finally, group 6 received **3ba** 80 mg/kg only.

**Figure 4:**
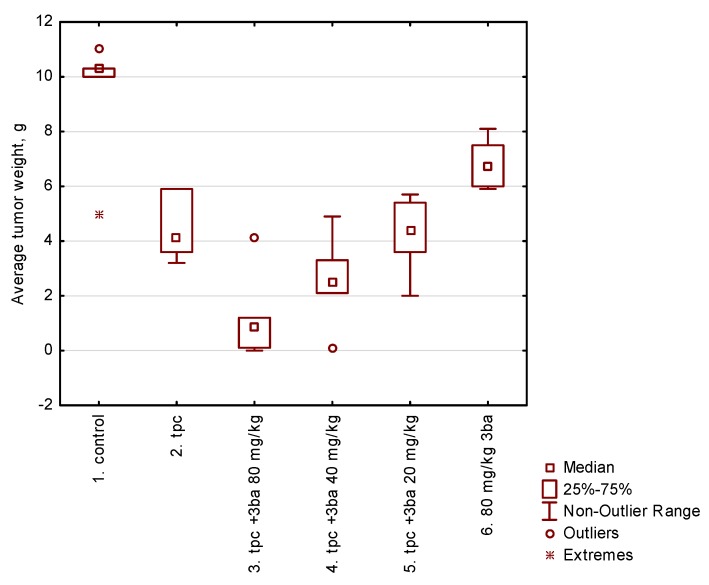
Box plot of the **3ba** influence on the antitumor effect of tpc against Krebs-2 carcinoma with intraperitoneal administration. P_1–2_ = 0.002; P_1–3_ = 0.00013; P_2–3_ = 0.04. The differences between group 2 and groups 4–6 are not significant.

The combined use of **3ba** at a maximum concentration of 80 mg/kg with tpc (group 3) led to a significant decrease in the weight of the ascitic tumor compared to the use of only tpc (Figure 4). The **3ba** dose of 40 mg/kg also caused a decrease in ascites weight, although the difference between groups 2 and 4 was not significant. The dose of **3ba** 20 mg/kg co-administered with tpc, as well as the use of **3ba** (80 mg/kg) in the absence of tpc, did not affect tumor growth.

**Figure 5:**
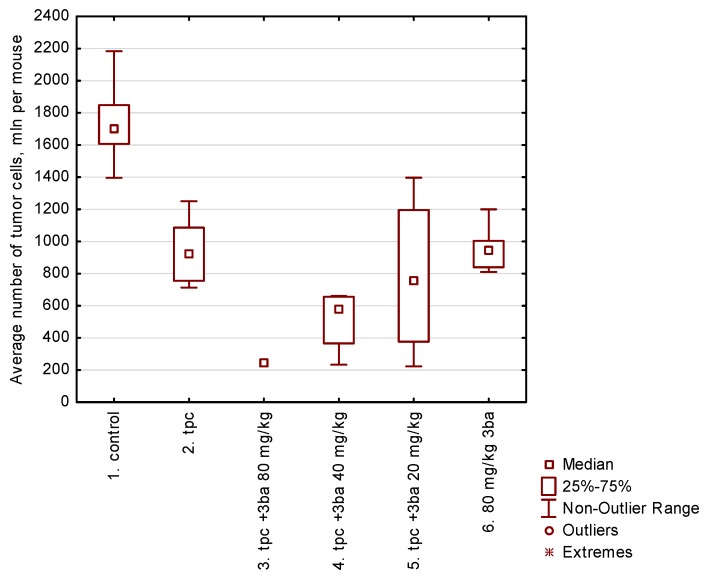
A box plot of the **3ba** influence on the number of tumor cells in ascites.

The number of tumor cells in ascites (Figure 5) in the control group (1750 million per mouse) and in group 2, which received only tpc (950 mln), was significantly different, i.e., by half, p = 0.005. The size of ascites in group 3 (tpc + 80 mg/kg **3ba**) was very small; we managed to extract ascitic fluid to determine the number of cells in only one mouse; the number of tumor cells in this mouse was 250 mln. Differences in other groups are not significant.

**Figure 6:**
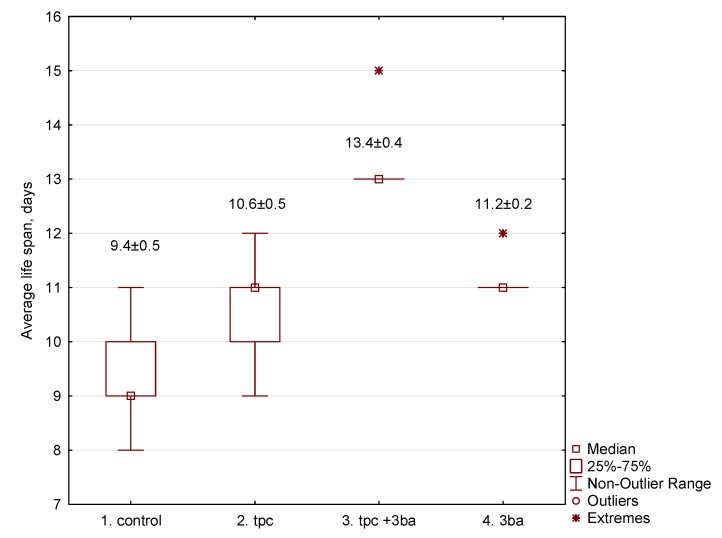
The influence of **3ba** in combination with tpc on the lifespan of mice. The numbers above the boxes indicate the average lifespan in the group.

We then examined the effect of **3ba** in combination with tpc on the lifespan of mice. C57BL/6 mice were intraperitoneally inoculated with the ascites variant of Lewis carcinoma. Group 1: control without treatment; group 2: tpc 0.5 mg/kg intraperitoneally; group 3: tpc and **3ba** intraperitoneally 120 mg/kg; and group 4: **3ba** 120 mg/kg only. The results are given in Figure 6.

When using a combination of tpc and **3ba**, a significant increase in lifespan was noted by 26% (p = 0.0065) compared with mice receiving only tpc, and by 42% compared with the control group (p = 0.0002). Monotherapy with tpc or **3ba** at selected doses prolonged the life of experimental animals unreliably, i.e., by 13–19%, *р* > 0.05.

### 2.3. In Silico

#### 2.3.1. Molecular Modeling

**Figure 7:**
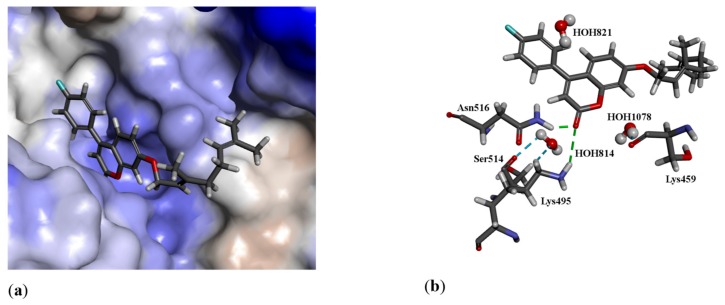
The docked configuration of **3ba** in the binding site of Tdp1 as predicted using the ChemPLP scoring function. (**a**) The protein surface is rendered. The ligand occupies the binding pocket. Blue depicts a hydrophilic region with a partial positive charge on the surface; brown depicts hydrophobic region with a partial negative charge and grey shows neutral areas. (**b**) Hydrogen bonds are shown as green lines between the ligand and residues Lys495 and Asn516. The water molecules also form hydrogen bonds with Ser514 and Lys459.

The 19 compounds were docked into the binding site of Tdp1 (PDB ID: 6DIE, resolution 1.78 Å) [66] with three water molecules (HOH 814, 821 and 1078). It has been shown that keeping these crystalline water molecules improves the prediction quality of the docking scaffold [45]. The modeling shows that all the ligands have a plausible binding mode and good scores with the four scoring functions used, i.e., Astex Statistical Potential (ASP) [67], improved Piecewise Linear Potential (ChemPLP) [68], ChemScore (CS) [69,70] and GoldScore (GS) [71]; the results are given in Table S2, Supplementary Information. Considering **3ba**, one of the most active compounds, the coumarin moiety occupies the hydrophilic binding region, which contains amino acids such as threonine and glutamic acid, whilst the alkene side chain occupies the hydrophobic region formed by isoleucine, leucine, and phenylalanine. The carbonyl on the benzopyrone group forms hydrogen bonds with the amine side chain groups of Lys495 and Asn516. The predicted binding mode of **3ba** is shown in Figure 7.

#### 2.3.2. Chemical Space

The calculated molecular descriptors (MW (molecular weight), log *P* (water-octanol partition coefficient), HD (hydrogen bond donors), HA (hydrogen bond acceptors), PSA (polar surface area), and RB (rotatable bonds)) are given in Table S3. The log *P* values range from 4.4 and 6.3, lying between the drug-like and Known Drug Space (KDS), while the HD and PSA values are within the lead-like space (for the definition of lead-like, drug-like, and KDS regions, see [72] and Table S4). The molecular weight of the ligands is between 326.4 and 453.4 g·mol^−1^, falling in the drug-like chemical space. The main issue with these ligands is their relatively high lipophilicity, with Log *P* values reaching into the KDS.

The Known Drug Indexes (KDIs) of each ligand were calculated to gauge the balance of the molecular descriptor of the ligands (Table S5, Supplementary Information). This method is based on the statistical analysis of drugs in clinical use (KDS) and a weighted index for each of the six molecular descriptors used; both the summation (KDI_2a_) and multiplication (KDI_2b_) methods were used [73]. The KDI_2a_ values range from 4.7 to 5.3, with a theoretical maximum of 6 and an average of 4.08 for known drugs. KDI_2b_ ranges from 0.2 to 0.5, with a theoretical maximum of 1 and a KDS average of 0.18. This indicates that the majority of the ligands are well balanced. The most potent ligand **3ba** has KDI_2a_ of 4.90 and KDI_2b_ of 0.27, while drugs with high bioavailability (>50%) have average KDI_2a_ of 4.43 and KDI_2b_ of 0.21, which shows that **3ba** has a very good balance of physicochemical properties for bioavailability. 

## 3. Materials and Methods

### 3.1. Chemistry Section

General Information. Reagents and solvents were purchased from commercial suppliers (Sigma-Aldrich, Acros) and used as received. GC-MS: *Agilent 7890A* gas chromatograph equipped with a quadrupole mass spectrometer *Agilent 5975C* as a detector; quartz column HP-5MS (copolymer 5%–diphenyl–95%–dimethylsiloxane) of length 30 m, internal diameter 0.25 mm and stationary phase film thickness 0.25 µm. Optical rotation: polAAr 3005 spectrometer. ^1^H and ^13^C NMR: *Bruker DRX-500* apparatus at 500.13 MHz (^1^H) and 125.76 MHz (^13^C) and *Bruker Avance—III 600* apparatus at 600.30 MHz (^1^H) and 150.95 MHz (^13^C), *J* in Hz; structure determinations by analyzing the ^1^H NMR spectra, including ^1^H–^1^H double resonance spectra and ^1^H–^1^H 2D homonuclear correlation, *J*-modulated ^13^C NMR spectra (JMOD), and ^13^C–^1^H 2D heteronuclear correlation with one-bond (C–H COSY, ^1^*J*(C,H) = 160 Hz, HSQC, ^1^*J*(C,H) = 145 Hz) and long-range spin-spin coupling constants (COLOC, ^2,3^*J*(C,H) = 10 Hz, HMBC, ^2,3^*J*(C,H) = 7 Hz). HR-MS: DFS Thermo Scientific spectrometer in a full scan mode (15–500 m/z, 70 eV electron impact ionization, direct sample administration). 

Spectral and analytical investigations were carried out at the Multi-Access Chemical Research Center of Siberian Branch of Russian Academy of Sciences. All product yields are given for pure compounds purified by recrystallization from ethanol or isolated by column chromatography (SiO_2_; 60–200 μ; *Macherey-Nagel*). The purity of the target compounds was determined by GC-MS methods. All of the target compounds reported in this paper have purities of no less than 95%.

#### 3.1.1. Synthesis of Compounds **5b–d**

General procedure. To a stirred mixture of sodium hydride (3 mol equiv), washed with hexane (3 × 15 mL), and diethyl carbonate (4 mol equiv) in 50 mL of tetrahydrofuran (THF), the corresponding substituted acetophenone (1 mol equiv) was added dropwise over 30 min. The reaction mixture was refluxed for 4 h, and then poured into ice water, acidified with 5 mL of glacial acetic acid, and extracted with EtOAc (3 × 100 mL). The combined organic phase was washed with saturated sodium bicarbonate, brine, and water, and then dried over anhydrous Na_2_SO_4_ and evaporated in vacuo. The crude products were purified by silica gel column chromatography eluting with dichloromethane to afford **5b–d**. The yields of **5b, 5c,** and **5d** were 80%, 89%, and 92%, respectively.

#### 3.1.2. Synthesis of Compounds **6a–d**

Syntheses were carried out from resorcinol **4** and appropriate β-keto esters (**5a–d**) in accordance with [46]. Conc. H_2_SO_4_ (2 mL, 37.6 mmol) was added dropwise to cooled (0–5 °C) solution of resorcinol **4** (2.3 g, 21 mmol) and appropriate β-keto esters (**5a–d**) (21 mmol) in dry ethanol (5 mL) with vigorously stirring. The reaction mixture was stirred at room temperature for 15 min, and then heated at 60 °C until it congealed. It was then left overnight at room temperature. Finally, it was poured into ice water (50 mL). The resulting solid was filtered off and crystallized from ethanol–water. The yields of **6a, 6b, 6c,** and **6d** were 81%, 79%, 73%, and 63% respectively.

#### 3.1.3. Synthesis of Compounds **8a–d**

(+)-Myrtenal was synthesized according to the procedure [46] by the oxidation of (+)-α-pinene using t-BuOOH/SeO_2_ system with a 57% yield. (−)- The (+)-myrtenols were synthesized from the corresponding aldehydes via reduction to alcohols with NaBH_4_, as described above. NaBH_4_ (10.3 mmol) was added to a cooled (0–5 °C) solution of 10.3 mmol of the appropriate aldehyde in methanol (20 mL), and the reaction mixture was stirred for 3 h at room temperature. Then, 5% aqueous HCl was added to reach a pH of 4–5. The solvent was distilled off and the product was extracted using ether and dried with Na_2_SO_4_. The solvent was evaporated; the resulting alcohols (58% and 54% yields) were used in the synthesis without purification.

Bromides **8a–d** were synthesized from geraniol, (−)-nopol, and (−)- and (+)-myrtenols via the aforementioned reaction with PBr_3_. PBr_3_ (8.9 mmol) was added to a cooled (0–5 °C) solution of the corresponding monoterpenoid alcohols (26.7 mmol) in dry ether (30 mL), and the reaction mixture was stirred for 2 h at room temperature. Saturated aqueous NaHCO_3_ was added, and the product was extracted with ether. The extracts were washed with brine, dried with Na_2_SO_4_, and evaporated. Compounds **8a**, **8c**, and **8d** (with yields of 91%, 55%, and 60%, respectively) were sufficiently pure and used for the next step without purification. The compound **8b** was purified by column chromatography on SiO_2_, eluent–hexane (yield 24%).

#### 3.1.4. Synthesis of Compounds **3aa**–**3da**, **3ab**–**3db**, **3ac**–**3dc**, **3ad**–**3dd**, and **10a**, **c**, **d**

Compounds **3aa–3da, 3ab–3db, 3ac–3dc, 3ad–3dd, and 10a, c, d** were synthesized from coumarins **6a–d** and the corresponding bromides **8a–d, 9** using DBU and DMF.

DBU (1.0 mmol) and corresponding bromide **8a–d, 9** (0.75 mmol) were added to compound **6a–d** (0.5 mmol) in dry DMF (5 mL) at room temperature under stirring. The reaction mixture was stirred at room temperature for 15 min and then heated at 60 °C for 5 h. H_2_O (15 mL) was added and the product was extracted with ethyl acetate. The extracts were washed with brine, dried with Na_2_SO_4_, and evaporated. The products **3aa–3da, 3ab–3db, 3ac–3dc, 3ad–3dd, and 10a, c, d** were isolated in the individual form **a**) by recrystallization from ethanol; or **b**) by column chromatography on silica gel using eluent–hexane, a solution containing from 25 to 100% ethyl acetate in hexane, and ethanol.

*(E)-7-(3,7-Dimethylocta-2,6-dienyloxy)-4-phenyl-2H-chromen-2-one****3aa.*** Yield 56%, method **a**. M.p. 54 °C. HRMS: 374.1879 [M]^+^; calcd. 374.1877 (C_25_H_26_O_3_)^+^. ^1^H NMR (CDCl_3_,*δ* ppm, *J*, Hz): 1.58 (br.s, 3H, CH_3_-24), 1.64 (m, 3H, all *J* < 1.5, CH_3_-23), 1.74 (m, 3H, all *J* < 1.5, CH_3_-25), 2.03-2.15 (m, 4H, 2H-19, 2H-20), 4.60 (d, 2H, *J*_16,17_ = 6.6, 2H-16), 5.06 (tm, 1H, *J*_21,20_ = 6.8, other *J* < 1.5, H-21), 5.45 (tm, 1H, *J*_17,16_ = 6.6, other *J* < 1.5, H-17), 6.18 (s, 1H, H-3), 6.77 (dd, 1H, *J*_7,6_ = 8.9, *J*_7,9_ = 2.5, H-7), 6.87 (d, 1H, *J*_9,7_ = 2.5, H-9), 7.34 (d, 1H, *J*_6,7_ = 8.9, H-6), 7.39-7.43 (m, 2H, H-11, H-15), 7.46-7.51 (m, 3H, H-12, H-13, H-14). ^13^C NMR (*δ* ppm, CDCl_3_): 155.85 (s, C-1), 161.12 (s, C-2), 111.61 (d, C-3), 155.70 (s, C-4), 112.28 (s, C-5), 127.75 (d, C-6), 112.81 (d, C-7), 161.99 (s, C-8), 101.75 (d, C-9), 135.52 (s, C-10), 128.23 (d, C-11, C-15), 128.66 (d, C-12, C-14), 129.40 (d, C-13), 65.38 (t, C-16), 118.34 (d, C-17), 142.19 (s, C-18), 39.37 (t, C-19), 26.11 (t, C-20), 123.49 (d, C-21), 131.78 (s, C-22), 25.50 (k, C-23), 17.56 (k, C-24), 16.63 (k, C-25). 

*(E)-7-(3,7-Dimethylocta-2,6-dienyloxy)-4-(4-fluorophenyl)-2H-chromen-2-one***3ba**. Yield 35%, method **a**. M.p. 72 °C. HRMS: 392.1778 [M]^+^; calcd. 392.1782 (C_25_H_25_FO_3_)^+^. ^1^H NMR (CDCl_3_,*δ* ppm, *J*, Hz): 1.58 (s, 3H, CH_3_-24), 1.64 (s, 3H, CH_3_-23), 1.74 (s, 3H, CH_3_-25), 2.02-2.16 (m, 4H, 2H-19, 2H-20), 4.60 (d, 2H, *J*_16,17_ = 6.5, 2H-16), 5.06 (tm, 1H, *J*_21,20_ = 6.8, other *J* < 2, H-21), 5.45 (tm, 1H, *J*_17,16_ = 6.5, other *J* < 2, H-17), 6.16 (s, 1H, H-3), 6.78 (dd, 1H, *J*_7,6_ = 8.9, *J*_7,9_ = 2.5, H-7), 6.87 (d, 1H, *J*_9,7_ = 2.5, H-9), 7.19 (dd, 2H, ***J***_12,11_ = ***J***_14,15_ = 8.7, *J*_12(14),F_ = 8.7, H-12, H-14), 7.30 (d, 1H, *J*_6,7_ = 8.9, H-6), 7.41 (dd, 2H, *J*_11,12_ =*J*_15,14_ = 8.7, *J*_11(15),F_ = 5.3, H-11, H-15). ^13^C NMR (*δ* ppm, CDCl_3,_ *J*_C,F_, Hz): 155.86 (s, C-1), 160.98 (s, C-2), 111.77 (d, C-3), 154.66 (s, C-4), 112.17 (s, C-5), 127.51 (d, C-6), 112.95 (d, C-7), 162.12 (d, C-8), 101.84 (d, C-9), 131.52 (s, ^4^*J* = 3.4, C-10), 130.18 (d, ^3^*J* = 8.3, C-11, C-15), 115.89 (d, ^2^*J* = 21.4, C-12, C-14), 163.35 (s, ^1^*J* = 250.0, C-13), 65.43 (t, C-16), 118.29 (d, C-17), 142.30 (s, C-18), 39.39 (t, C-19), 26.13 (t, C-20), 123.49 (d, C-21), 131.82 (s, C-22), 25.51 (k, C-23), 17.57 (k, C-24), 16.64 (k, C-25). 

*(E)-4-(4-Bromophenyl)-7-(3,7-dimethylocta-2,6-dienyloxy)-2H-chromen-2-one***3ca**. Yield 40%, method **a**. M.p. 80 °C. HRMS: 452.0979 [M]^+^; calcd. 452.0982 (C_25_H_25_BrO_3_)^+^. ^1^H NMR (CDCl_3_,*δ* ppm, *J*, Hz): 1.58 (br.s, 3H, CH_3_-24), 1.64 (m, 3H, all *J* < 1.5, CH_3_-23), 1.74 (br.s, 3H, CH_3_-25), 2.03-2.14 (m, 4H, 2H-19, 2H-20), 4.59 (d, 2H, *J*_16,17_ = 6.6, 2H-16), 5.06 (tm, 1H, *J*_21,20_ = 6.7, other *J* ≤ 1.5, H-21), 5.44 (tm, 1H, *J*_17,16_ = 6.6, other *J* < 1.5, H-17), 6.16 (s, 1H, H-3), 6.78 (dd, 1H, *J*_7,6_ = 8.9, *J*_7,9_ = 2.5, H-7), 6.87 (d, 1H, *J*_9,7_ = 2.5, H-9), 7.28 (d, 1H, *J*_6,7_ = 8.9, H-6), 7.29 (br.d, 2H, *J*_11,12_ =*J*_15,14_ = 8.5, H-11, H-15), 7.63 (br.d, 2H, *J*_12,11_ = *J*_14,15_ = 8.5, H-12, H-14). ^13^C NMR (*δ* ppm, CDCl_3_): 155.82 (s, C-1), 160.92 (s, C-2), 111.65 (d, C-3), 154.52 (s, C-4), 111.83 (s, C-5), 127.43 (d, C-6), 112.99 (d, C-7), 162.13 (c, C-8), 101.78 (d, C-9), 134.31 (s, C-10), 129.84 (d, C-11, C-15), 131.98 (d, C-12, C-14), 123.87 (s, C-13), 65.39 (t, C-16), 118.16 (d, C-17), 142.38 (s, C-18), 39.37 (t, C-19), 26.08 (t, C-20), 123.45 (d, C-21), 131.84 (s, C-22), 25.54 (k, C-23), 17.59 (k, C-24), 16.65 (k, C-25). 

*(E)-7-(3,7-Dimethylocta-2,6-dienyloxy)-4-(4-methoxyphenyl)-2H-chromen-2-one***3da.** Yield 29%, method **b**. HRMS: 404.1980 [M]^+^; calcd. 404.1982 (C_26_H_28_O_4_)^+^. ^1^H NMR (CDCl_3_,*δ* ppm, *J*, Hz): 1.58 (s, 3H, CH_3_-24), 1.64 (m, 3H, all *J* < 2.0, CH_3_-23), 1.74 (m, 3H, all *J* < 1.5, CH_3_-25), 2.03-2.15 (m, 4H, 2H-19, 2H-20), 3.86 (s, 3H, CH_3_-26), 4.59 (d, 2H, *J*_16,17_ = 6.6, 2H-16), 5.06 (tm, 1H, *J*_21,20_ = 6.8, other *J* < 1.5, H-21), 5.45 (tm, 1H, *J*_17,16_ = 6.6, other *J* < 1.5, H-17), 6.16 (s, 1H, H-3), 6.78 (dd, 1H, *J*_7,6_ = 8.9, *J*_7,9_ = 2.5, H-7), 6.86 (d, 1H, *J*_9,7_ = 2.5, H-9), 7.00 (br.d, 2H, *J*_12,11_ = *J*_14,15_ = 8.7, H-12, H-14), 7.37 (br.d, 2H, *J*_11,12_ =*J*_15,14_ = 8.7, H-11, H-15), 7.41 (d, 1H, *J*_6,7_ = 8.9, H-6). ^13^C NMR (*δ* ppm, CDCl_3_): 155.85 (s, C-1), 161.34 (s, C-2), 111.08 (d, C-3), 155.40 (s, C-4), 112.39 (s, C-5), 127.78 (d, C-6), 112.74 (d, C-7), 161.87 (s, C-8), 101.70 (d, C-9), 127.74 (s, C-10), 129.73 (d, C-11, C-15), 114.12 (d, C-12, C-14), 160.60 (s, C-13), 65.34 (t, C-16), 118.31 (d, C-17), 142.21 (s, C-18), 39.38 (t, C-19), 26.10 (t, C-20), 123.48 (d, C-21), 131.80 (s, C-22), 25.52 (k, C-23), 17.57 (k, C-24), 16.63 (k, C-25), 55.28 (k, C-26).

*7-(2-((1R,5S)-6,6-Dimethylbicyclo[3 1.1]hept-2-en-2-yl)ethoxy)-4-phenyl-2H-chromen-2-one***3ab**. Yield 12%, method **b**. HRMS: 386.1872 [M]^+^; calcd. 386.1877 (C_26_H_26_O_3_)^+^. ${\left[\alpha \right]}_{589}^{22}$ = −20.0 (*c* = 0.53, CHCl_3_). ^1^H NMR (CDCl_3_,*δ* ppm, *J*, Hz): 0.81 (c, 3H, CH_3_-26), 1.16 (d, 1H, *J*_24a,24s_ = 8.6, H-24a), 1.26 (s, 3H, CH_3_-25), 2.05-2.11 (m, 2H, H-21, H-23), 2.20 (dm, 1H, *J*_20,20′_ = 17.6, other *J* < 3.5, H-20), 2.26 (dm, 1H, *J*_20′,20_ = 17.6, other *J* < 3.5, H-20′), 2.37 (ddd, 1H, *J*_24s,24a_ = 8.6, *J*_24s,21_ = *J*_24s,23_ = 5.6, H-24s), 2.40-2.52 (m, 2H, 2H-17), 3.98-4.06 (m, 2H, 2H-16), 5.32-5.36 (m, 1H, H-19), 6.18 (s, 1H, H-3), 6.74 (dd, 1H, *J*_7,6_ = 8.9, *J*_7,9_ = 2.5, H-7), 6.85 (d, 1H, *J*_9,7_ = 2.5, H-9), 7.34 (d, 1H, *J*_6,7_ = 8.9, H-6), 7.39-7.44 (m, 2H, H-11, H-15), 7.46-7.51 (m, 3H, H-12, H-13, H-14). ^13^C NMR (*δ* ppm, CDCl_3_): 155.91 (s, C-1), 161.11 (s, C-2), 111.63 (d, C-3), 155.71 (s, C-4), 112.30 (s, C-5), 127.80 (d, C-6), 112.63 (d, C-7), 162.07 (s, C-8), 101.50 (d, C-9), 135.54 (s, C-10), 128.25 (d, C-11, C-15), 128.68 (d, C-12, C-14), 129.42 (d, C-13), 66.90 (t, C-16), 36.07 (t, C-17), 143.89 (s, C-18), 118.99 (d, C-19), 31.27 (t, C-20), 40.64 (d, C-21), 37.97 (c, C-22), 45.79 (d, C-23), 31.54 (t, C-24), 26.18 (k, C-25), 21.08 (k, C-26).

*7-(2-((1R,5S)-6,6-Dimethylbicyclo[3.1.1]hept-2-en-2-yl)ethoxy)-4-(4-fluorophenyl)-2H-chromen-2-one***3bb**. Yield 37%, method **b**. HRMS: 404.1774 [M]^+^; calcd. 404.1782 (C_26_H_25_FO_3_)^+^. ${\left[\alpha \right]}_{589}^{22}$ = −17.4 (*c* = 0.78, CHCl_3_). ^1^H NMR (CDCl_3_,*δ* ppm, *J*, Hz): 0.81 (s, 3H, CH_3_-26), 1.16 (d, 1H, *J*_24a,24s_ = 8.6, H-24a), 1.26 (s, 3H, CH_3_-25), 2.06-2.10 (m, 2H, H-21, H-23), 2.20 (dm, 1H, *J*_20,20′_ = 17.7, H-20), 2.27 (dm, 1H, *J*_20′,20_ = 17.7, H-20′), 2.36 (ddd, 1H, *J*_24s,24a_ = 8.6, *J*_24s,21_ = *J*_24s,23_ = 5.6, H-24s), 2.40-2.51 (m, 2H, 2H-17), 4.02 (t, 2H, *J*_16,17_ = 7.0, 2H-16), 5.32-5.36 (m, 1H, H-19), 6.16 (s, 1H, H-3), 6.75 (dd, 1H, *J*_7,6_ = 8.9, *J*_7,9_ = 2.5, H-7), 6.84 (d, 1H, *J*_9,7_ = 2.5, H-9), 7.16-7.21 (m, 2H, *J*_12,11_ = *J*_14,15_ = 8.7, *J*_12(14),F_ =8.7, H-12, H-14), 7.30 (d, 1H, *J*_6,7_ = 8.9, H-6), 7.38-7.43 (m, 2H, *J*_11,12_ = *J*_15,14_ = 8.7, *J*_11(15),F_ = 5.3, H-11, H-15). ^13^C NMR (*δ* ppm, CDCl_3_, *J*_C,F_, Hz): 155.90 (s, C-1), 160.94 (s, C-2), 111.76 (d, C-3), 154.64 (s, C-4), 112.16 (s, C-5), 127.54 (d, C-6), 112.74 (d, C-7), 162.18 (s, C-8), 101.57 (d, C-9), 131.51 (s, ^4^*J* = 3.5, C-10), 130.18 (d, ^3^*J* = 8.3, C-11, C-15), 115.88 (d, ^2^*J* = 21.8, C-12, C-14), 66.93 (t, C-16), 36.05 (t, C-17), 143.86 (s, C-18), 119.02 (d, C-19), 31.27 (t, C-20), 40.64 (d, C-21), 37.97 (s, C-22), 45.79 (d, C-23), 31.54 (t, C-24), 26.17 (k, C-25), 21.08 (k, C-26).

*4-(4-Bromophenyl)-7-(2-((1R,5S)-6,6-dimethylbicyclo[3.1.1]hept-2-en-2-yl)ethoxy)-2H-chromen-2-one***3cb**. Yield 15%, method **b**. ${\left[\alpha \right]}_{589}^{26.6}$ = −17.9 (*c* = 0.58, CHCl_3_). HRMS: 464.0988 [M]^+^; calcd. 450.0982 (C_26_H_25_BrO_3_)^+^. ^1^H NMR (CDCl_3_,*δ* ppm, *J*, Hz): 0.80 (s, 3H, CH_3_-26), 1.15 (d, 1H, *J*_24a,24s_ = 8.6, H-24a), 1.25 (s, 3H, CH_3_-25), 2.06-2.09 (m, 2H, H-21, H-23), 2.19 (dm, 1H, *J*_20,20′_ = 17.6, H-20), 2.26 (dm, 1H, *J*_20′,20_ = 17.6, H-20′), 2.36 (ddd, 1H, *J*_24s,24a_ = 8.6, *J*_24s,21_ = *J*_24s,23_ = 5.6, H-24s), 2.40-2.51 (m, 2H, 2H-17), 3.98-4.04 (m, 2H, 2H-16), 5.33-5.36 (m, 1H, H-19), 6.16 (s, 1H, H-3), 6.75 (dd, 1H, *J*_7,6_ = 8.9, *J*_7,9_ = 2.5, H-7), 6.84 (d, 1H, *J*_9,7_ = 2.5, H-9), 7.28 (d, 1H, *J*_6,7_ = 8.9, H-6), 7.28-7.31 (m, 2H, *J*_11,12_ = *J*_15,14_ = 8.4, H-11, H-15), 7.61-7.64 (m, 2H, *J*_12,11_ = *J*_14,15_ = 8.4, H-12, H-14). ^13^C NMR (*δ* ppm, CDCl_3_): 155.86 (s, C-1), 160.87 (s, C-2), 111.65 (d, C-3), 154.49 (s, C-4), 111.81 (s, C-5), 127.44 (d, C-6), 112.79 (d, C-7), 162.19 (s, C-8), 101.50 (d, C-9), 134.30 (s, C-10), 129.84 (d, C-11, C-15), 131.97 (d, C-12, C-14), 123.86 (s, C-13), 66.87 (t, C-16), 36.01 (t, C-17), 143.78 (s, C-18), 119.03 (d, C-19), 31.23 (t, C-20), 40.54 (d, C-21), 37.95 (s, C-22), 45.65 (d, C-23), 31.51 (t, C-24), 26.14 (k, C-25), 21.08 (k, C-26).

*7-(2-((1R,5S)-6,6-Dimethylbicyclo[3.1.1]hept-2-en-2-yl)ethoxy)-4-(4-methoxyphenyl)-2H-chromen-2-one***3db**. Yield 65%, method **b**. HRMS: 415.1906 [M-H]^+^; calcd. 415.1904 (C_27_H_27_O_4_)^+^. ${\left[\alpha \right]}_{589}^{22}$ = −15.7 (*c* = 0.75, CHCl_3_). ^1^H NMR (CDCl_3_,*δ* ppm, *J*, Hz): 0.81 (s, 3H, CH_3_-27), 1.16 (d, 1H, *J*_25a,25s_ = 8.6, H-25a), 1.26 (s, 3H, CH_3_-26), 2.05-2.11 (m, 2H, H-21, H-24), 2.19 (dm, 1H, ^2^*J* = 17.6, H-21), 2.26 (dm, 1H, ^2^*J* = 17.6, H-21′), 2.36 (ddd, 1H, *J*_25s,25a_ = 8.6, *J*_25s,22_ = *J*_25s,24_ = 5.6, H-25s), 2.40-2.51 (m, 2H, 2H-18), 3.86 (s, 3H, CH_3_-16), 4.02 (t, 2H, *J*_17,18_ = 7.0, H-17), 5.32-5.36 (m, 1H, H-20), 6.15 (s, 1H, H-3), 6.75 (dd, 1H, *J*_7,6_ = 8.9, *J*_7,9_ = 2.5, H-7), 6.83 (d, 1H, *J*_9,7_ = 2.5, H-9), 7.01 (br.d, 2H, *J*_12,11_ = *J*_14,15_ = 8.7, H-12, H-14), 7.37 (br.d, 2H, *J*_11,12_ = *J*_15,14_ = 8.7, H-11, H-15), 7.40 (d, 1H, *J*_6,7_ = 8.9, H-6). ^13^C NMR (*δ* ppm, CDCl_3_): 155.93 (s, C-1), 161.28 (s, C-2), 111.12 (d, C-3), 155.38 (s, C-4), 112.43 (s, C-5), 127.81 (d, C-6), 112.54 (d, C-7), 161.97 (s, C-8), 101.49 (d, C-9), 127.79 (s, C-10), 129.74 (d, C-11, C-15), 114.16 (d, C-12, C-14), 160.64 (s, C-13), 55.30 (k, C-16), 66.88 (t, C-17), 36.07 (t, C-18), 143.90 (s, C-19), 118.97 (d, C-20), 31.27 (t, C-21), 40.64 (d, C-22), 37.97 (s, C-23), 45.79 (d, C-24), 31.54 (t, C-25), 26.17 (k, C-26), 21.08 (k, C-27).

*7-(((1R,5S)-6,6-Dimethylbicyclo[3.1.1]hept-2-en-2-yl)methoxy)-4-phenyl-2H-chromen-2-one***3ac**. Yield 40%, method **a**. M.p. 106 °C. HRMS: 372.1717 [M]^+^; calcd. 372.1720 (C_25_H_24_O_3_)^+^. ${\left[\alpha \right]}_{589}^{27.3}$ = −25.33 (*c* = 1.02, EtOH). ^1^H NMR (CDCl_3_,*δ* ppm, *J*, Hz): 0.82 (s, 3H, CH_3_-25), 1.18 (d, 1H, ^2^*J* = 8.7, H-23a), 1.28 (s, 3H, CH_3_-24), 2.08-2.14 (m, 1H, H-20), 2.20 (ddd, 1H, *J*_22,20_ = *J*_22,23s_ = 5.6, *J*_22,18_ = 1.4, H-22), 2.26 (dm, 1H, ^2^*J* = 18.1, H-19), 2.33 (dm, 1H, ^2^*J* = 18.1, H-19′), 2.40 (ddd, 1H, ^2^*J* = 8.7, *J*_23s__,20_= *J*_23s__,22_ = 5.6, H-23s), 4.44 (dm, 1H, ^2^*J* = 12.4, other *J* ≤ 2.0, H-16), 4.47 (dm, 1H, ^2^*J* = 12.4, other *J* ≤ 2.0, H-16′), 5.61-5.64 (m, 1H, H-18), 6.19 (s, 1H, H-3), 6.77 (dd, 1H, *J*_7,6_ = 8.9, *J*_7,9_ = 2.5, H-7), 6.89 (d, 1H, *J*_9,7_ = 2.5, H-9), 7.33 (d, 1H, *J*_6,7_ = 8.9, H-6), 7.40-7.43 (m, 2H, H-11, H-15), 7.47-7.51 (m, 3H, H-12, H-13, H-14). ^13^C NMR (*δ* ppm, CDCl_3_): 155.81 (s, C-1), 161.24 (s, C-2), 111.65 (d, C-3), 155.76 (s, C-4), 112.32 (s, C-5), 127.68 (d, C-6), 112.94 (d, C-7), 162.14 (s, C-8), 102.00 (d, C-9), 135.55 (s, C-10), 128.27 (d, C-11, C-15), 128.68 (d, C-12, C-14), 129.42 (d, C-13), 71.12 (t, C-16), 142.92 (s, C-17), 121.34 (d, C-18), 31.18 (t, C-19), 40.71 (d, C-20), 38.00 (s, C-21), 43.11 (d, C-22), 31.40 (t, C-23), 26.02 (k, C-24), 20.97 (k, C-25).

*7-(((1R,5S)-6,6-Dimethylbicyclo[3.1.1]hept-2-en-2-yl)methoxy)-4-(4-fluorophenyl)-2H-chromen-2-one***3bc**. Yield 53%, method **b**. ${\left[\alpha \right]}_{589}^{22.0}$ = −15.6 (*c* = 0.68, CHCl_3_). HRMS: 390.1624 [M]^+^; calcd. 390.1626 (C_25_H_23_FO_3_)^+^. ^1^H NMR (CDCl_3_,*δ* ppm, *J*, Hz): 0.81 (s, 3H, CH_3_-25), 1.17 (d, 1H, *J*_23a,23s_ = 8.7, H-23a), 1.28 (s, 3H, CH_3_-24), 2.11 (ddtd, 1H, *J*_20,22_ = *J*_20,23s_ = 5.6, *J*_20,19_ = 2.9, *J*_20,18_ = 1.3, H-20), 2.20 (ddd, 1H, *J*_22,20_ = *J*_22,23s_ = 5.6, *J*_22,18_ = 1.4, H-22), 2.26 (dm, 1H, *J*_19,19_^,^ = 18.0, other *J* < 3.5, H-19), 2.33 (dm, 1H, *J*_19′,19_ = 18.0, other *J* < 3.5, H-19′), 2.40 (ddd, 1H, *J*_23s,23a_ = 8.7, *J*_23s,20_= *J*_23s,22_ = 5.6, H-23s), 4.41-4.49 (m, 2H, 2H-16), 5.61-5.64 (m, 1H, H-18), 6.16 (s, 1H, H-3), 6.78 (dd, 1H, *J*_7,6_ = 8.9, *J*_7,9_ = 2.5, H-7), 6.88 (d, 1H, *J*_9,7_ = 2.5, H-9), 7.16-7.21 (m, 2H, *J*_12,11_ = *J*_14,15_ = 8.8, *J*_12(14),F_ = 8.6, H-12, H-14), 7.29 (d, 1H, *J*_6,7_ = 8.9, H-6), 7.38-7.43 (m, 2H, *J*_11,12_ = *J*_15,14_ = 8.8, *J*_11(15),F_ = 5.3, H-11, H-15). ^13^C NMR (*δ* ppm, CDCl_3_, *J*_C,F_, Hz): 155.80 (s, C-1), 161.02 (s, C-2), 111.78 (d, C-3), 154.66 (s, C-4), 112.18 (s, C-5), 127.42 (d, C-6), 113.03 (d, C-7), 162.23 (s, C-8), 102.07 (d, C-9), 131.52 (s, ^4^*J* = 3.5, C-10), 130.19 (d, ^3^*J* = 8.3, C-11, C-15), 115.88 (d, ^2^*J* = 21.8, C-12, C-14), 71.14 (t, C-16), 142.86 (s, C-17), 121.39 (d, C-18), 31.18 (t, C-19), 40.69 (d, C-20), 38.00 (s, C-21), 43.09 (d, C-22), 31.39 (t, C-23), 26.00 (k, C-24), 20.97 (k, C-25).

*4-(4-Bromophenyl)-7-(((1R,5S)-6,6-dimethylbicyclo[3.1.1]hept-2-en-2-yl)methoxy)-2H-chromen-2-one***3cc**. Yield 36%, method **a**. M.p. 130 °C. HRMS: 450.0826 [M]^+^; calcd. 450.0825 (C_25_H_23_O_3_Br)^+^. ${\left[\alpha \right]}_{589}^{26.4}$ = −13.9 (*c* = 0.52, CHCl_3_). ^1^H NMR (CDCl_3_,*δ* ppm, *J*, Hz): 0.80 (s, 3H, CH_3_-25), 1.16 (d, 1H, *J*_23a,23s_ = 8.7, H-23a), 1.28 (s, 3H, CH_3_-24), 2.08-2.12 (m,1H, H-20), 2.19 (ddd, 1H, *J*_22,20_ = *J*_22,23s_ = 5.6, *J*_22,18_ = 1.3, H-22), 2.25 (dm, 1H, *J*_19,19_^,^ = 18.0, other *J* < 3.5, H-19), 2.33 (dm, 1H, *J*_19_^,^_,19_ = 18.0, other *J* < 3.5, H-19′), 2.40 (ddd, 1H, *J*_23s,23a_ = 8.7, *J*_23s,20_= *J*_23s,22_ = 5.6, H-23s), 4.43-4.48 (m, 2H, 2H-16), 5.60-5.63 (m, 1H, H-18), 6.16 (s, 1H, H-3), 6.78 (dd, 1H, *J*_7,6_ = 8.9, *J*_7,9_ = 2.5, H-7), 6.88 (d, 1H, *J*_9,7_ = 2.5, H-9), 7.27 (d, 1H, *J*_6,7_ = 8.9, H-6), 7.30 (d, 2H, *J*_11,12_ = *J*_15,14_ = 8.5, H-11, H-15), 7.63 (d, 2H, *J*_12,11_ = *J*_14,15_ = 8.5, H-12, H-14). ^13^C NMR (*δ* ppm, CDCl_3_): 155.76 (s, C-1), 160.97 (s, C-2), 111.67 (d, C-3), 154.53 (s, C-4), 111.85 (s, C-5), 127.33 (d, C-6), 113.10 (d, C-7), 162.25 (s, C-8), 102.02 (d, C-9), 134.32 (s, C-10), 129.85 (d, C-11, C-15), 131.98 (d, C-12, C-14), 123.87 (s, C-13), 71.12 (t, C-16), 142.77 (s, C-17), 121.43 (d, C-18), 31.15 (t, C-19), 40.60 (d, C-20), 37.99 (s, C-21), 43.00 (d, C-22), 31.37 (t, C-23), 25.98 (k, C-24), 20.96 (k, C-25).

*7-(((1R,5S)-6,6-Dimethylbicyclo[3.1.1]hept-2-en-2-yl)methoxy)-4-(4-methoxyphenyl)-2H-chromen-2-one***3dc**. Yield 55%, method **b**. M.p. 124 °C. ${\left[\alpha \right]}_{589}^{30.6}$ = −17.3 (*c* = 0.82, CHCl_3_). HRMS: 402.1820 [M]^+^; calcd. 402.1827 (C_26_H_26_O_4_)^+^. ^1^H NMR (CDCl_3_,*δ* ppm, *J*, Hz): 0.81 (s, 3H, CH_3_-26), 1.17 (d, 1H, *J*_24a,24s_ = 8.7, H-24a), 1.28 (s, 3H, CH_3_-25), 2.10 (ddtd, 1H, *J*_21,23_ = *J*_21,24s_ = 5.6, *J*_21,20_ = 2.8, *J*_21,19_ = 1.3, H-21), 2.20 (ddd, 1H, *J*_23,21_ = *J*_23,24s_ = 5.6, *J*_23,19_ = 1.4, H-23), 2.25 (dm, 1H, *J*_20,20_^,^ = 18.0, other *J* < 3.0, H-20), 2.33 (dm, 1H, *J*_20′,20_ = 18.0, other *J* < 3.5, H-20′), 2.40 (ddd, 1H, *J*_24s,24a_ = 8.7, *J*_24s,21_= *J*_24s,23_ = 5.6, H-24s), 3.85 (s, 3H, CH_3_-16), 4.41-4.49 (m, 2H, 2H-17), 5.60-5.63 (m, 1H, H-19), 6.14 (s, 1H, H-3), 6.77 (dd, 1H, *J*_7,6_ = 8.9, *J*_7,9_ = 2.5, H-7), 6.86 (d, 1H, *J*_9,7_ = 2.5, H-9), 7.00 (br.d, 2H, *J*_12,11_ = *J*_14,15_ = 8.8, H-12, H-14), 7.36 (br.d, 2H, *J*_11,12_ = *J*_15,14_ = 8.8, H-11, H-15), 7.39 (d, 1H, *J*_6,7_ = 8.9, H-6). ^13^C NMR (*δ* ppm, CDCl_3_): 155.80 (s, C-1), 161.30 (s, C-2), 111.11 (d, C-3), 155.36 (s, C-4), 112.41 (s, C-5), 127.67 (d, C-6), 112.79 (d, C-7), 161.99 (s, C-8), 101.96 (d, C-9), 127.77 (s, C-10), 129.72 (d, C-11, C-15), 114.13 (d, C-12, C-14), 160.62 (s, C-13), 55.27 (k, C-16), 71.06 (t, C-17), 142.91 (s, C-18), 121.26 (d, C-19), 31.15 (t, C-20), 40.67 (d, C-21), 37.97 (s, C-22), 43.07 (d, C-23), 31.37 (t, C-24), 25.99 (k, C-25), 20.94 (k, C-26).

*7-(((1S,5R)-6,6-Dimethylbicyclo[3.1.1]hept-2-en-2-yl)methoxy)-4-phenyl-2H-chromen-2-one***3ad**. Yield 46%, method **b**. ${\left[\alpha \right]}_{589}^{26.6}$= +23.33 (*c*=1.02, EtOH). HRMS: 372.1718 [M]^+^; calcd. 372.1720 (C_25_H_24_O_3_)^+^. The ^1^H and ^13^C NMR spectra of **3ad** correspond to the spectra of the enantiomer **3ac**.

*7-(((1S,5R)-6,6-Dimethylbicyclo[3.1.1]hept-2-en-2-yl)methoxy)-4-(4-fluorophenyl)-2H-chromen-2-one***3bd**. Yield 35%, method **a**. M.p. 129 °C. HRMS: 390.1628 [M]^+^; calcd. 390.1626 (C_25_H_23_FO_3_)^+^. ${\left[\alpha \right]}_{589}^{22.0}$ = +22.3 (CHCl_3_, *c* = 0.53). The ^1^H and ^13^C NMR spectra of **3bd** correspond to the spectra of the enantiomer **3bc**.

*4-(4-Bromophenyl)-7-(((1S,5R)-6,6-dimethylbicyclo[3.1.1]hept-2-en-2-yl)methoxy)-2H-chromen-2-one****3cd***. Yield 39%, method **a**. M.p. 138 °C. HRMS: 450.0825 [M]^+^; calcd. 450.0820 (C_25_H_23_BrO_3_)^+^. ${\left[\alpha \right]}_{589}^{26.7}$ = +22.4 (*c* = 0.58, CHCl_3_). The ^1^H and ^13^C NMR spectra of **3cd** correspond to the spectra of the enantiomer **3cc**.

*7-(((1S,5R)-6,6-Dimethylbicyclo[3.1.1]hept-2-en-2-yl)methoxy)-4-(4-methoxyphenyl)-2H-chromen-2-one****3dd***. Yield 33%, method **a**. M.p. 112 °C. HRMS: 402.1823 [M]^+^; calcd. 402.1827 (C_26_H_26_O_4_)^+^. ${\left[\alpha \right]}_{589}^{22.0}$ = +20.8 (*c* = 0.72, CHCl_3_). The ^1^H and ^13^C NMR spectra of **3dd** correspond to the spectra of the enantiomer **3dc**.

*7-(Benzyloxy)-4-phenyl-2H-chromen-2-one***10a**. Yield 42%, method **b**. M.p. 92 °C. HRMS: 328.1093 [M]^+^; calcd. 328.1094 (C_22_H_16_O_3_)^+^. ^1^H NMR (CDCl_3_,*δ* ppm, *J*, Hz): 5.13 (s, 2H, 2H-16), 6.19 (s, 1H, H-3), 6.85 (dd, 1H, *J*_7,6_ = 8.9, *J*_7,9_ = 2.5, H-7), 6.94 (d, 1H, *J*_9,7_ = 2.5, H-9), 7.31-7.35 (m, 1H, H-20), 7.35–7.44 (m, 7H, H-6, H-11, H-15, H-18, H-19, H-21, H-22), 7.46-7.52 (m, 3H, H-12, H-13, H-14). ^13^C NMR (*δ* ppm, CDCl_3_): 155.80 (s, C-1), 161.00 (s, C-2), 111.86 (d, C-3), 155.62 (s, C-4), 112.62 (s, C-5), 127.88 (d, C-6), 112.80 (d, C-7), 161.71 (s, C-8), 102.09 (d, C-9), 135.44 (s, C-10), 128.23 (d, C-11, C-15), 128.68 (d, C-12, C-14), 129.44 (d, C-13), 70.39 (t, C-16), 135.67 (s, C-17), 127.35 (d, C-18, C-22), 128.62 (d, C-19, C-21), 128.23 (d, C-20).

*7-(Benzyloxy)-4-(4-bromophenyl)-2H-chromen-2-one***10c**. Yield 25%, method **a**. M.p. 128 °C. HRMS: 406.0204 [M]^+^; calcd. 406.0199 (C_22_H_15_BrO_3_)^+^. ^1^H NMR (CDCl_3_,*δ* ppm, *J*, Hz): 5.13 (s, 2H, 2H-16), 6.17 (s, 1H, H-3), 6.86 (dd, 1H, *J*_7,6_ = 8.9, *J*_7,9_ = 2.5, H-7), 6.94 (d, 1H, *J*_9,7_ = 2.5, H-9), 7.29 (dm, 2H, *J*_11,12_ = *J*_15,14_ = 8.5, H-11, H-15), 7.31 (d, 1H, *J*_6,7_ = 8.9, H-6), 7.32-7.35 (m, 1H, H-20), 7.37-7.43 (m, 4H, H-18, H-19, H-21, H-22), 7.63 (dm, 2H, *J*_12,11_ = *J*_14,15_ = 8.5, H-12, H-14). ^13^C NMR (*δ* ppm, CDCl_3_): 155.79 (s, C-1), 160.81 (s, C-2), 111.92 (d, C-3), 154.46 (s, C-4), 112.18 (s, C-5), 127.56 (d, C-6), 113.02 (d, C-7), 161.86 (s, C-8), 102.14 (d, C-9), 134.24 (s, C-10), 129.83 (d, C-11, C-15), 132.01 (d, C-12, C-14), 123.92 (s, C-13), 70.42 (t, C-16), 135.54 (s, C-17), 127.38 (d, C-18, C-22), 128.66 (d, C-19, C-21), 128.30 (d, C-20).

*7-(Benzyloxy)-4-(4-methoxyphenyl)-2H-chromen-2-one***10d**. Yield 34%, method **b**. M.p. 143 °C. HRMS: 358.1198 [M]^+^; calcd. 358.1200 (C_23_H_18_O_4_)^+^. ^1^H NMR (CDCl_3_,*δ* ppm, *J*, Hz): 3.86 (s, 3H, CH_3_-16), 5.12 (s, 2H, 2H-17), 6.16 (s, 1H, H-3), 6.85 (dd, 1H, *J*_7,6_ = 8.9, *J*_7,9_ = 2.5, H-7), 6.93 (d, 1H, *J*_9,7_ = 2.5, H-9), 7.01 (dm, 2H, *J*_12,11_ = *J*_14,15_ = 8.7, H-12, H-14), 7.30-7.35 (m, 1H, H-21), 7.35–7.45 (m, 7H, H-6, H-11, H-15, H-19, H-20, H-22, H-23). ^13^C NMR (*δ* ppm, CDCl_3_): 155.82 (s, C-1), 161.18 (s, C-2), 111.34 (d, C-3), 155.30 (s, C-4), 112.75 (s, C-5), 127.90 (d, C-6), 112.72 (d, C-7), 161.61 (s, C-8), 102.07 (d, C-9), 127.68 (s, C-10), 129.72 (d, C-11, C-15), 114.16 (d, C-12, C-14), 160.65 (s, C-13), 55.29 (k, C-16), 70.37 (t, C-17), 135.70 (s, C-18), 127.36 (d, C-19, C-23), 128.61 (d, C-20, C-22), 128.22 (d, C-21).

### 3.2. Biology Section

*Real-Time Detection of Tdp1 Activity.* The Tdp1 activity measurements were carried out as described [64]. Briefly, Tdp1-biosensor with a final concentration of 50 nM was incubated in a volume of 200 μL containing buffer (50 mM Tris-HCl pH8.0, 50 mM NaCl, 7 mM β-mercaptoethanol) supplemented with purified 1.3 nM Tdp1. The reactions were incubated in a POLARstar OPTIMA fluorimeter, BMG LABTECH, GmbH, to measure fluorescence every 1 min (Ex485/Em520 nm). Tdp1 inhibition was calculated by comparing the rate of increase in fluorescence in the presence of the compound to that of DMSO control wells. IC_50_ values were determined using a 6-point concentration response curve. The data were imported into the MARS Data Analysis 2.0 program (BMG LABTECH), and the slope during the linear phase (here data from 0 to 7 min) was calculated.

*Cell Culture Assays*. Tumor cells from human mammary adenocarcinoma cell line MCF-7 and cervical cancer cell line HeLa (~2000 cells per well) were incubated for 24 h at 37 °C in IMDM medium (5% CO_2_), and then treated with the synthesized derivatives. After 72 h of cell incubation, the relative amount of alive cells was determined using standard colorimetric MTT test [74] or EZ4U Cell Proliferation and Cytotoxicity Assay (Biomedica, Austria), as per the manufacturer’s protocols.

*Binding Assay***.** Synthetic DNA encoding human Tdp1 (residues 149-608) was cloned into pET-28a (+) (GenScript), which was then transformed into *Escherichia coli* BL21 (DE3) for recombinant protein production. Protein production was induced with 1 mM IPTG at 28 °C with overnight incubation. Purification of Tdp1 was performed using affinity and size exclusion chromatography. Intrinsic protein fluorescence was measured using PerkinElmer EnSpire Multimode Reader. The Tdp1 concentration was 10 µM, and the compound concentrations were 25 µM, 50 µM, 75 µM, 100 µM, 150 µM, and 250 µM. The buffer was composed of 20 mM Tris and 250 mM NaCl, pH 8. The excitation wavelength was 280 nm and the intrinsic fluorescence was measured at 350 nm. Compound control was performed using the buffer and compound only. The total volume per well was 30 µL. Dissociation constants (*K*_D_) were calculated using the following formula, that takes nonspecific binding into account.

$$I=\frac{Imax\;\times \left[L_{T}\right]}{K_{D}+\left[L_{T}\right]}+Ns\left[L_{T}\right]$$

In this formula, I indicates changes in fluorescence intensity from the titration, Imax indicates the maximum fluorescence intensity change, [*L*ᴛ] is the titration ligand concentration, and Ns is the non-specific term. Non-linear curve fitting was conducted using SigmaPlot 13.0 (Systat Software, San Jose, CA, USA). Experiments were conducted in triplicate and the errors shown are standard derivations.

*Lab animals.* Thre-e to four-month-old male and female C57Bl/6 mice from the breeding colony of the Institute of Cytology and Genetics, SB RAS, were used in the study. The animals were kept on sawdust in plastic cages with 5–7 mice per cage, with free access to ground food (“Laboratorkorm”, Moskow, Russia) and tap water. All experiments were performed in accordance with protocols approved by the Animal Care and Use Committee of the Institute of Cytology and Genetics. Also, all experimental procedures were performed in accordance with the Directive 2010/63/EU for animal experiments.

*Tumor models.* The experimental tumor used was Lewis Lung Carcinoma (LLC) and Krebs-2. The animals were treated with tpc and the Tdp1 inhibitor **3ba** two days after tumor transplantation. The tumor was transplanted into the muscles of the thigh by 0.2 million cells per mouse. Tpc (Sindan Pharma SRL, Romania) was administered intraperitoneally at a single dose of 0.5 mg/kg; Tdp1 inhibitor **3ba** was administrated intraperitoneally at a single dose of 20, 40, or 80 mg/kg (for Krebs-2), or 120 mg/kg (for LLC) in 15% dimethyl sulfoxide (DMSO)–10% Tween-80 suspension in water (0.2 mL of suspension per mouse) simultaneously with tpc. Control mice were injected with a DMSO-Tween-80 mixture into the stomach.

The antitumor effect was assessed by the size and weight of the solid tumors at 18 days after transplantation. For estimations of daily gain in volume, the tumor nodules were periodically measured with a caliper.

*Statistical analysis.* The experimenter measuring and calculating the primary animal data (tumor size, lifespan) was blinded. After unblinding, the animal data were statistically processed using oneway ANOVA. Post-hoc testing was completed using Turkey’s Honestly Significant Difference (HSD). *p* < 0.05 was considered to be statistically significant. The statistical package STATISTICA version 12.5 was used for analysis. All results are expressed as mean ± SEM.

### 3.3. Modeling Section

*Molecular modeling and chemical space.* The compounds were docked against the crystal structure of Tdp1 (PDB ID: 6DIE, resolution 1.78 Å) [75], which was obtained from the Protein Data Bank (PDB) [76,77]. The Scigress version FJ 2.6 program [78] was used to prepare the crystal structure for docking, i.e., the hydrogen atoms were added, and the cocrystallized ligand benzene-1,2,4-tricarboxylic acid was removed, as well as crystallographic water molecules, except HOH 814, 821, and 1078. The Scigress software suite was also used to build the inhibitors, and the MM2 [77] force field was used to optimize the structures. The docking centre was defined as the position of a carbon on the ring of benzene-1, 2, 4-tricarboxylic acid (*x* = −6.052, *y* = −14.428, *z* = 33.998) with 10 Å radius. Fifty docking runs were allowed for each ligand with the default search efficiency (100%). The basic amino acids lysine and arginine were defined as protonated. Furthermore, aspartic and glutamic acids were assumed to be deprotonated. The GoldScore(GS) [71] and ChemScore (CS) [69,70], ChemPLP (Piecewise Linear Potential) [68], and ASP (AstexStatistical Potential) [67] scoring functions were implemented to validate the predicted binding modes and relative energies of the ligands using the GOLD v5.4.1 software suite (The Cambridge Crystallographic Data Centre, Cambridge, UK). The QikProp 3.2 [79] software package (Schrödinger, New York, USA) was used to calculate the molecular descriptors of the molecules; the reliability of this method has been established for the calculated descriptors [80].

## 4. Conclusions

Overall, we reported the synthesis and evaluation of novel Tdp1 inhibitors that combine the arylcoumarin and monoterpenoid moieties. Our results found that these compounds are good Tdp1 inhibitors with IC_50_ in the submicromolar or low submicromolar ranges. Compound **3ba** showed a significant increase in the antitumor effect of tpc on Krebs-2 ascites in an in vivo tumor model. In addition, these compounds presented the good physicochemical properties required for oral bioavailability, making them good candidates for further development. Thus, this type of arylcoumarin-monoterpenoid hybrids represents an excellent starting point for the further development of adjuvant therapy against cancer in combination with Top 1 poisons.

## Supplementary Materials

Supplementary materials can be found at https://www.mdpi.com/1422-0067/21/1/126/s1.

## Abbreviations

Tdp1	Tyrosyl-DNA phosphodiesterase 1
Top1	topoisomerase 1
CPTs	camptothecin derivatives
tpc	topotecan
