# Supplementary material for: Promising New Inhibitors of Tyrosyl-DNA Phosphodiesterase I (Tdp 1) Combining 4-Arylcoumarin and Monoterpenoid Moieties as Components of Complex Antitumor Therapy
Source: Int J Mol Sci. 2019 Dec 23;21(1):126. doi: 10.3390/ijms21010126 (PMC6982354; doi:10.3390/ijms21010126)
Supplement: Supplementary file 1 [file ijms-21-00126-s001.pdf]

SUPPLEMENTARY INFORMATION

New Promising Inhibitors of Tyrosyl-DNA Phosphodiesterase I (Tdp 1) Combining 4-Arylcoumarin and Monoterpenoid Moieties as Components of Complex Antitumor Therapy

Tatyana M. Khomenko, Alexandra L. Zakharenko, Arina A. Chepanova, Ekaterina S. Ilyina, Olga D. Zakharova, Vasily I. Kaledin, Valeriy P. Nikolin, Nelly A. Popova, Dina V. Korchagina, Jóhannes Reynisson, Raina Chand, Daniel M. Ayine-Tora, Jinal Patel, Ivanhoe K. H. Leung, Konstantin P. Volcho, Nariman F. Salakhutdinov, Olga I. Lavrik

NMR ^{1}H and ^{13}C spectra of the compounds **3aa - 3da, 3ab - 3db, 3ac - 3dc and 10a, c, d.**

Table S1. The Tdp1 inhibitory activities of compounds **3aa - 3dd** and **10a, c, d**.

Table S2. The scores for the 19 ligands docked against Tdp1.

Table S3. The molecular descriptor values for the 19 compounds.

Table S4. Definition of lead-like, drug-like and Known drug space (KDS) in terms of molecular descriptors. The values given are the maxima for each descriptor for the volumes of chemical space used.

Table S5. The calculated KDI indexes for the 19 ligands.

Supplementary Figure S1. (a) Change in intrinsic fluorescence intensity of Tdp1 (10 μM) upon the addition of compound **3ba** (25 μM, 50 μM, 75 μM, 100 μM, 150 μM and 250 μM).

Compound **3aa**

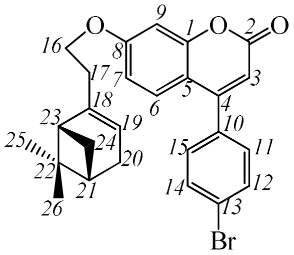

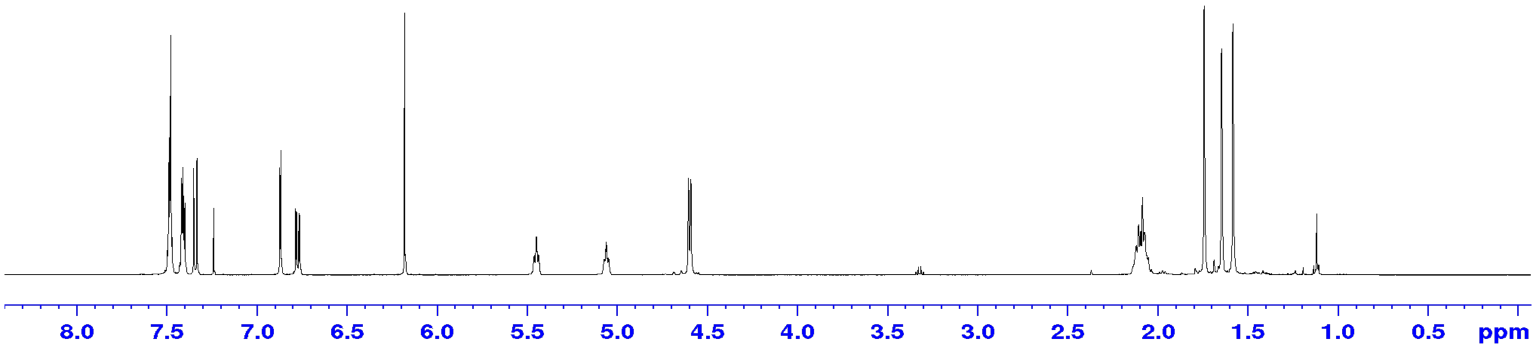

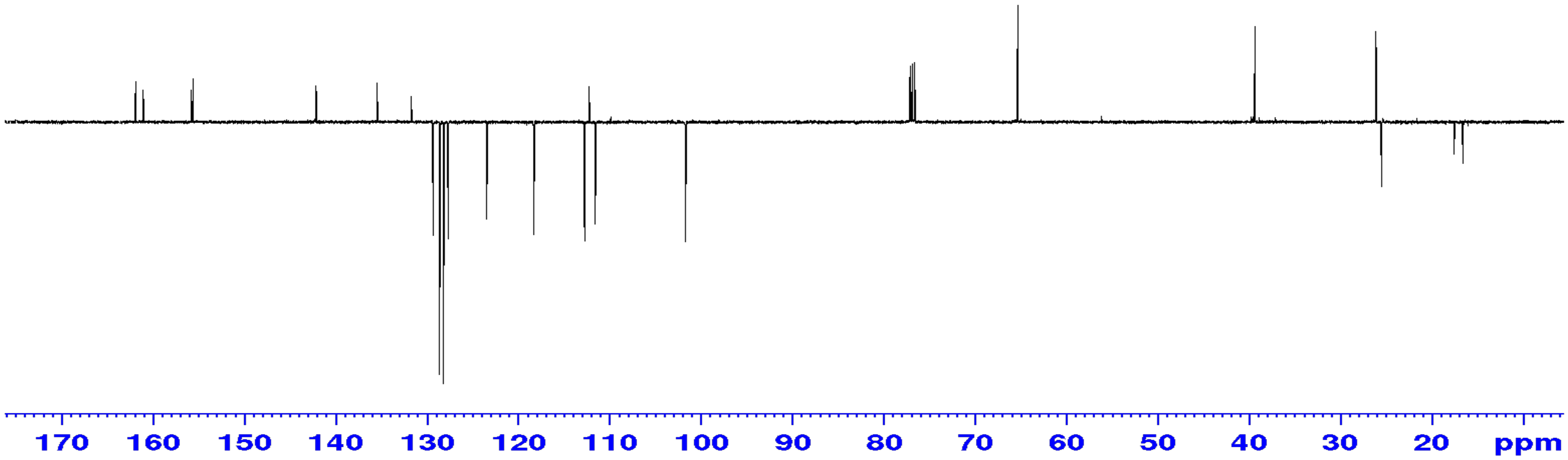

Compound **3ba**

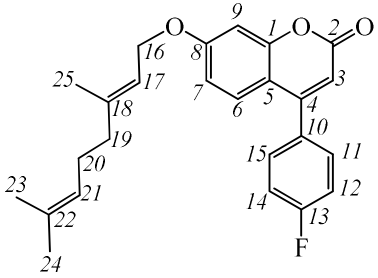

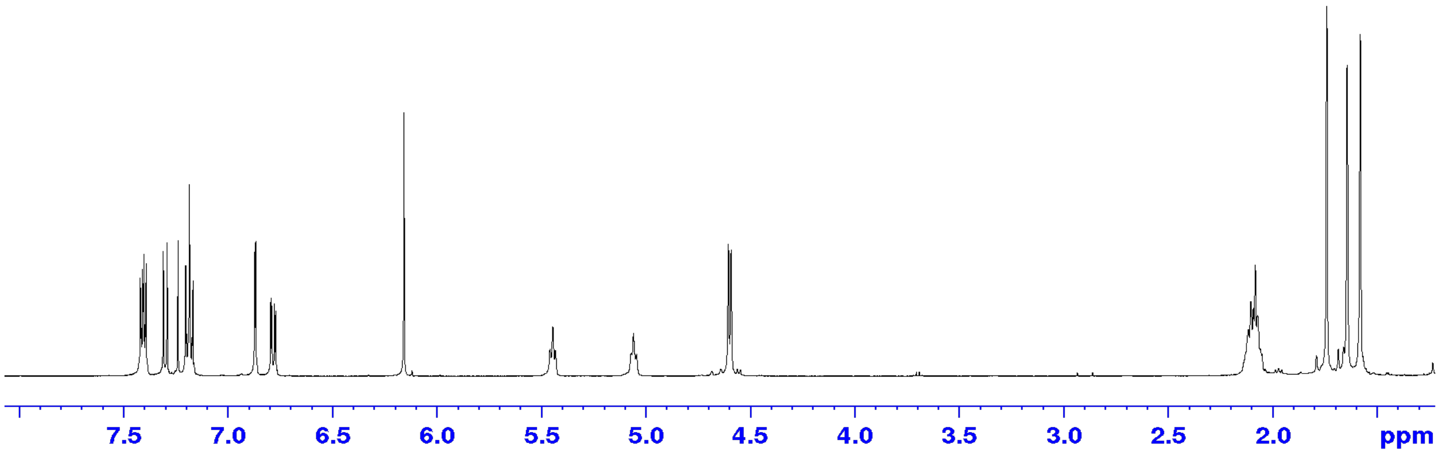

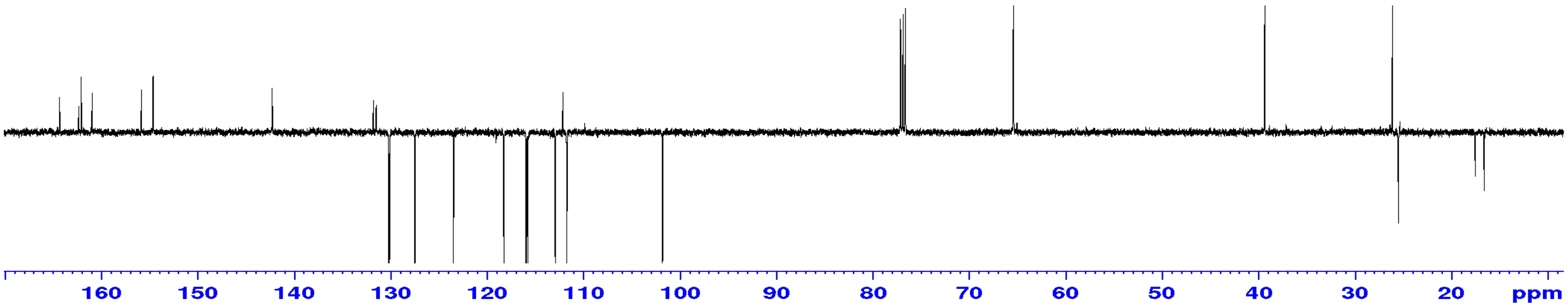

Compound **3ca**

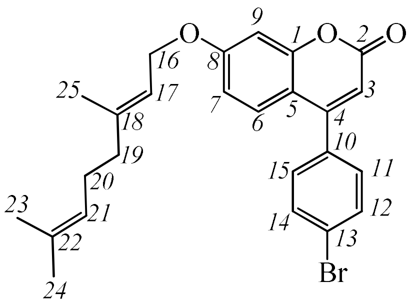

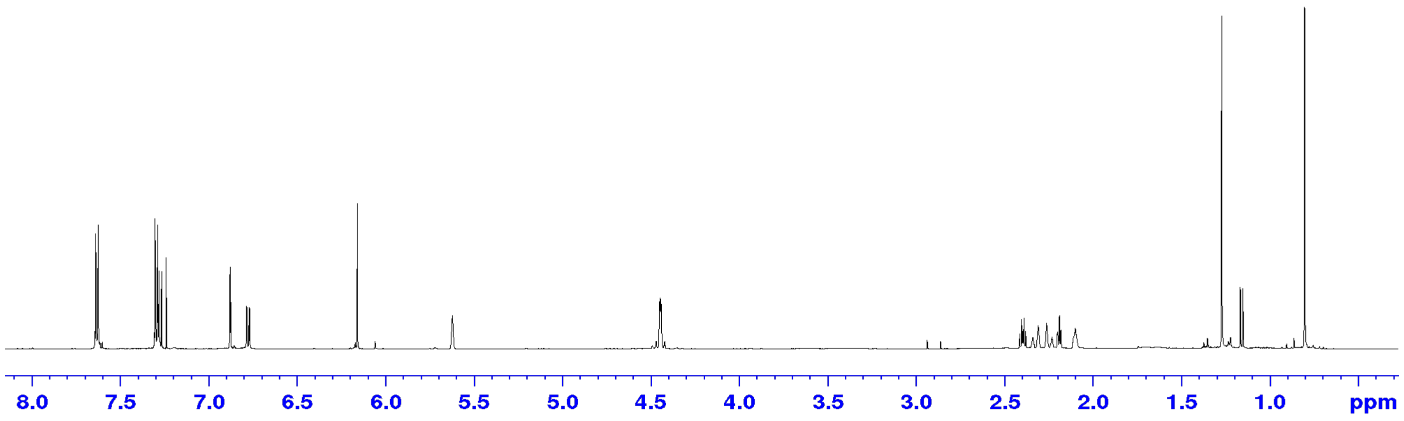

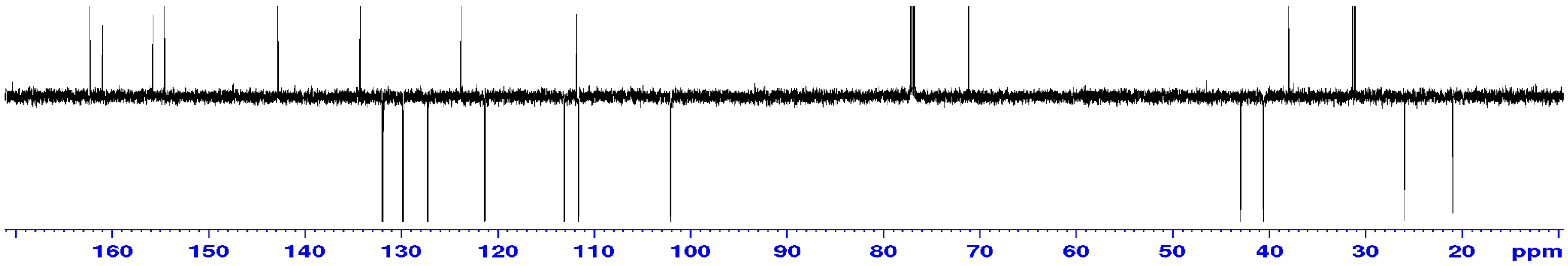

Compound **3da**

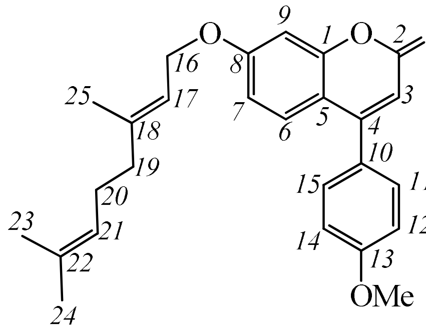

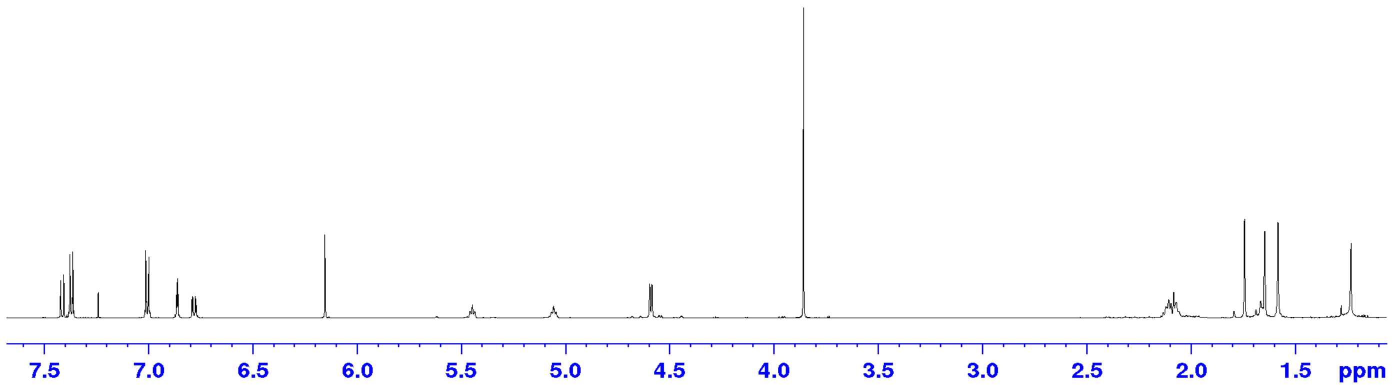

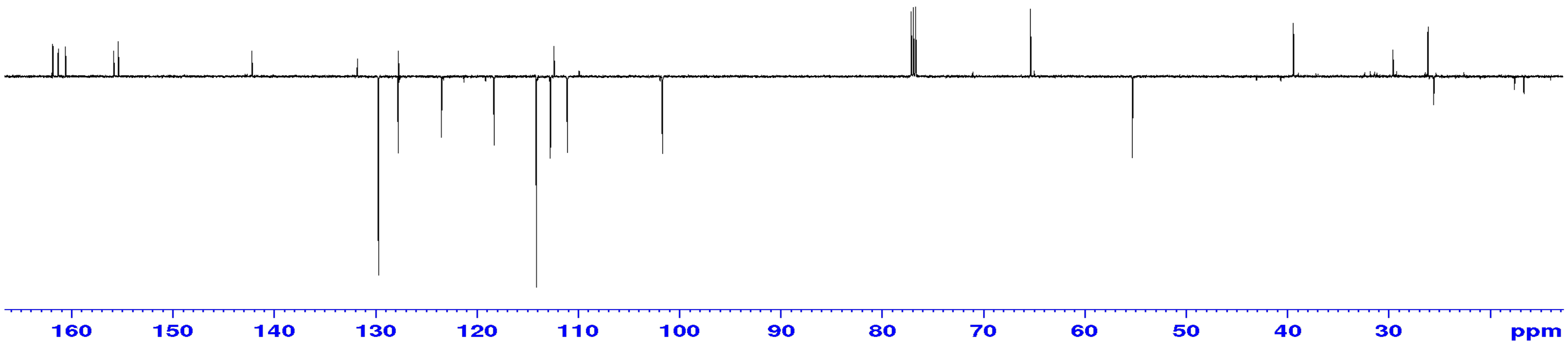

Compound **3ab**

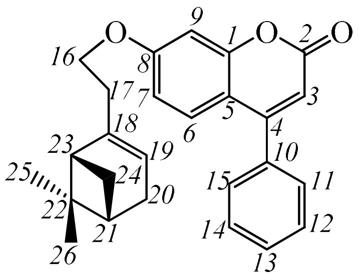

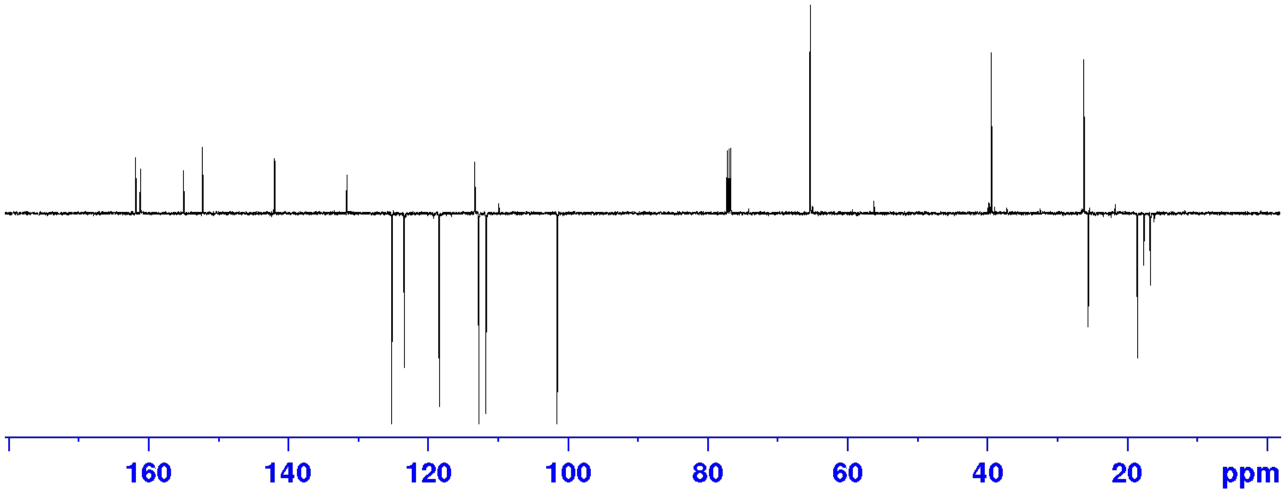

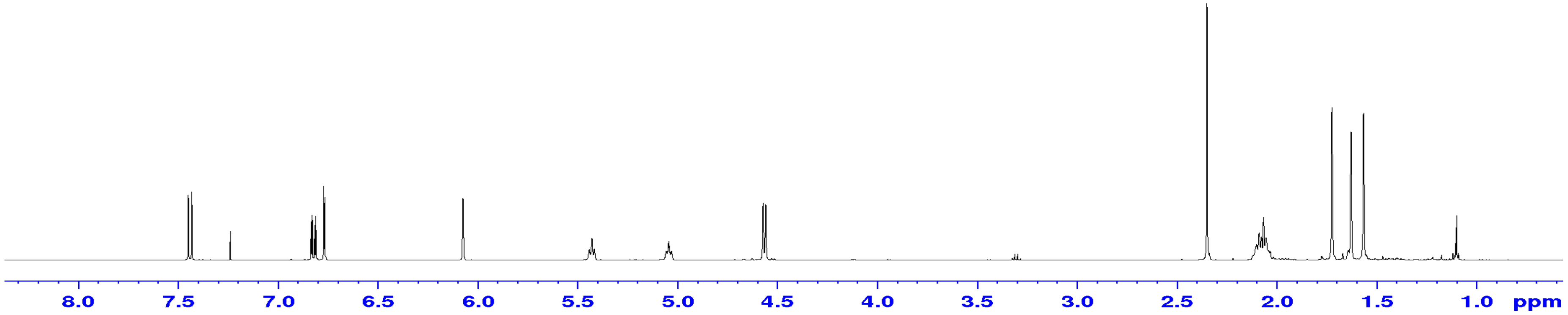

Compound **3bb**

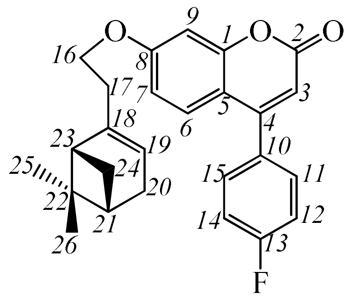

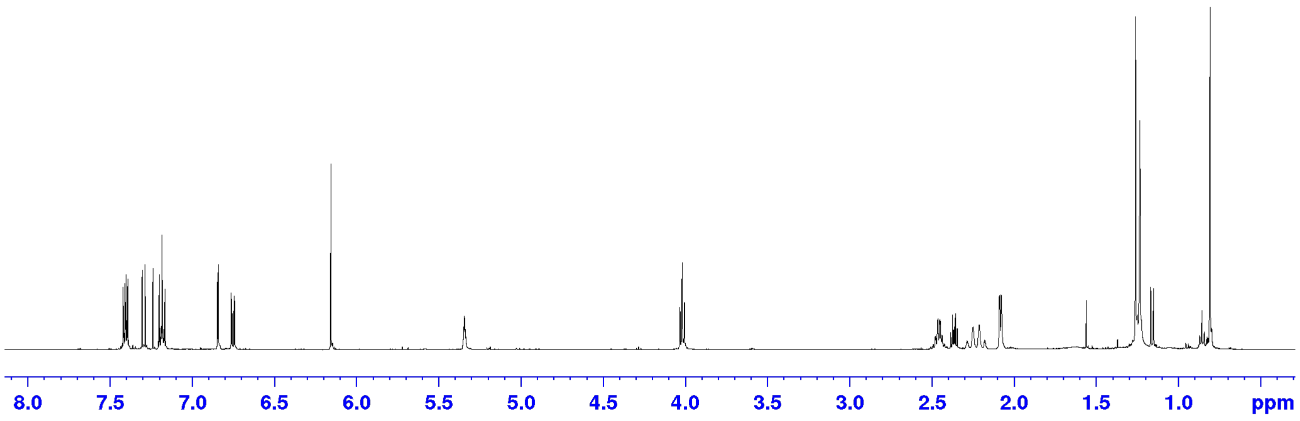

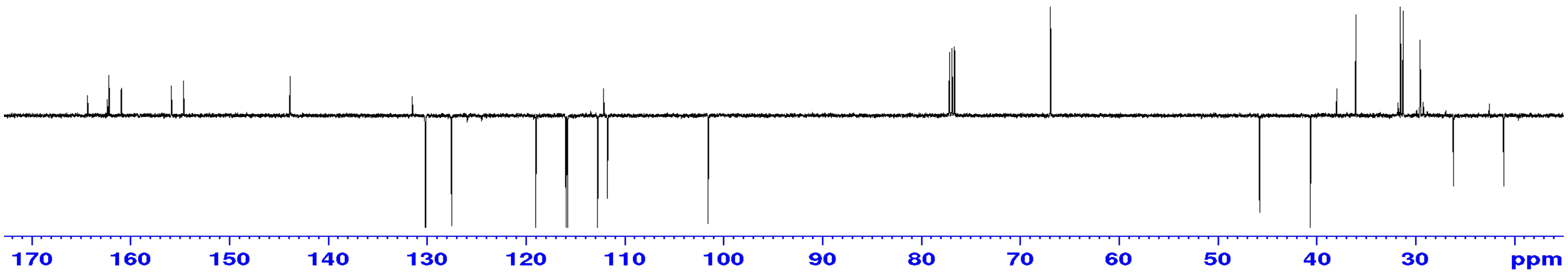

Compound **3cb**

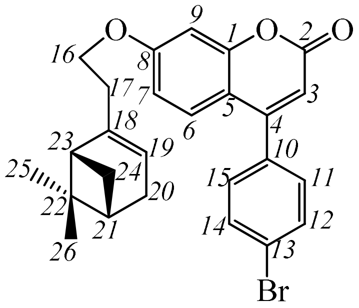

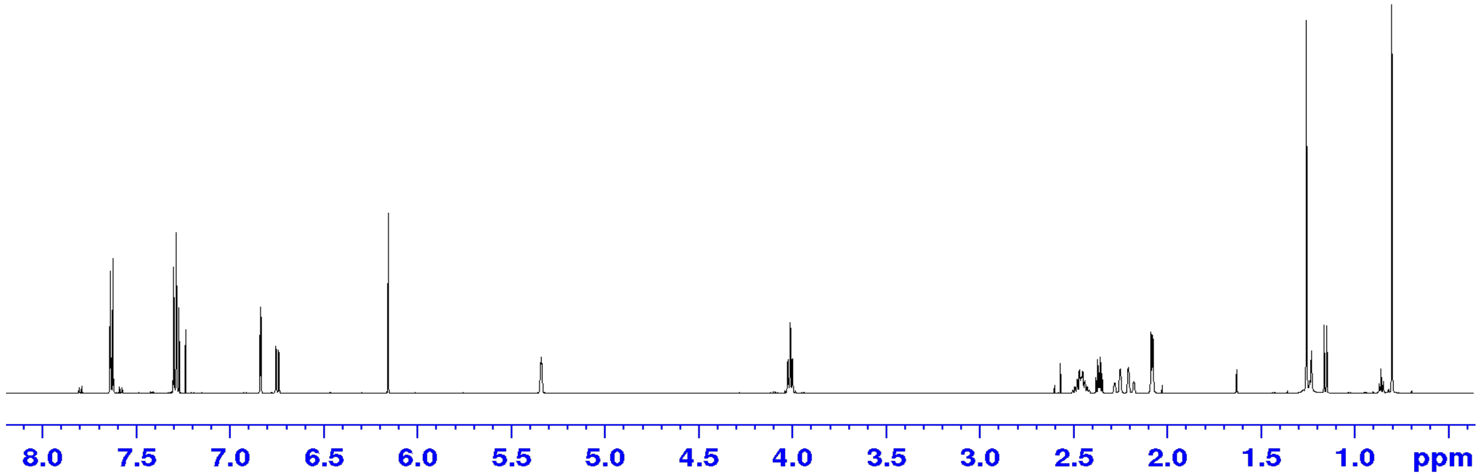

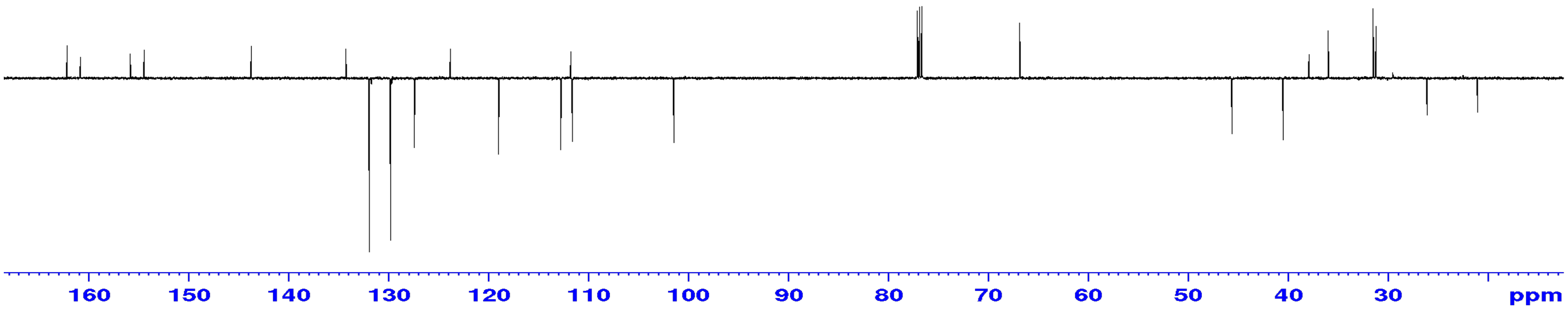

Compound **3db**

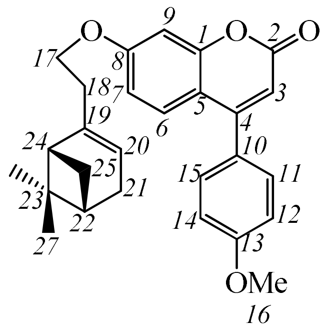

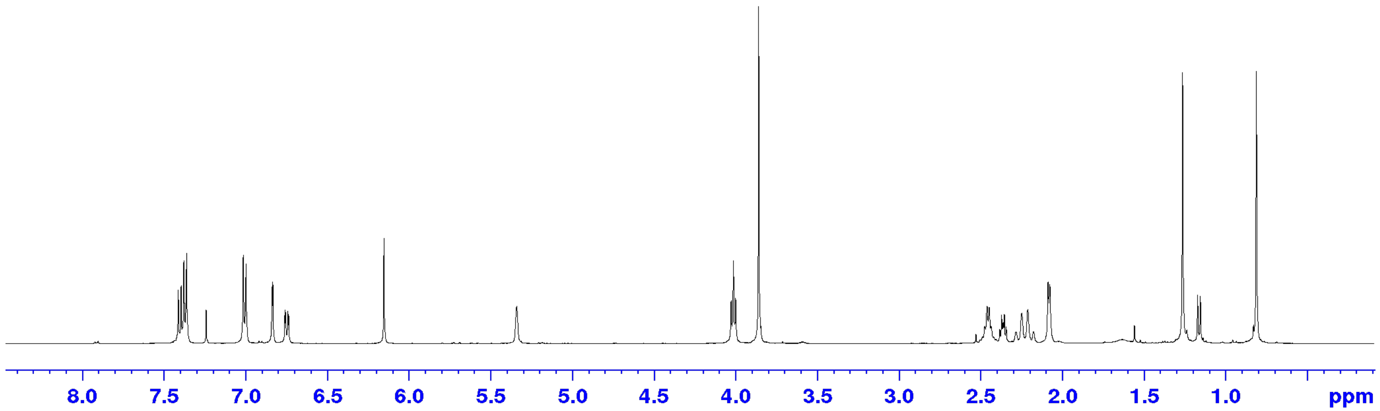

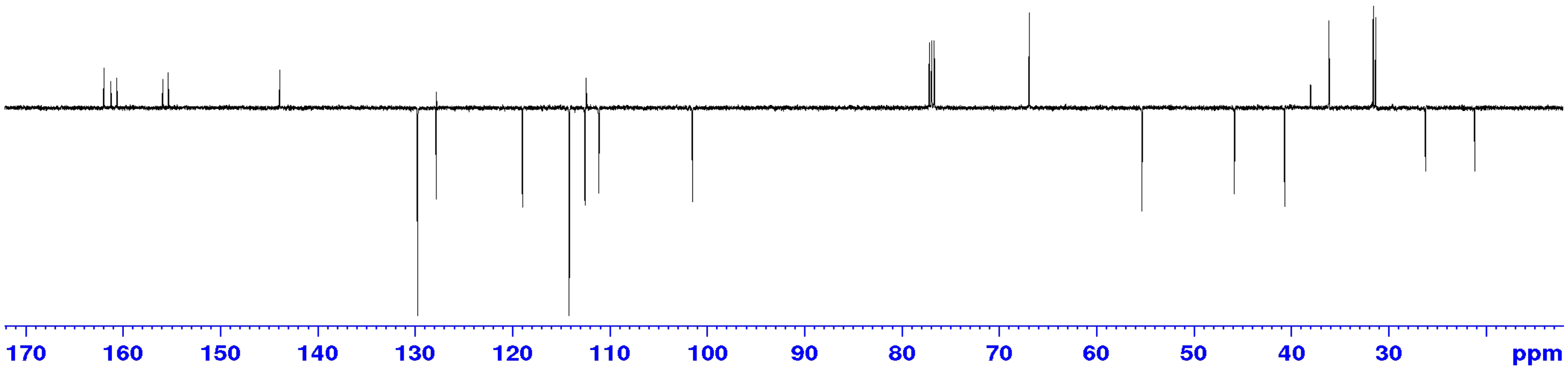

Compound **3ac**

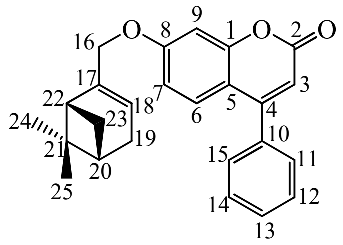

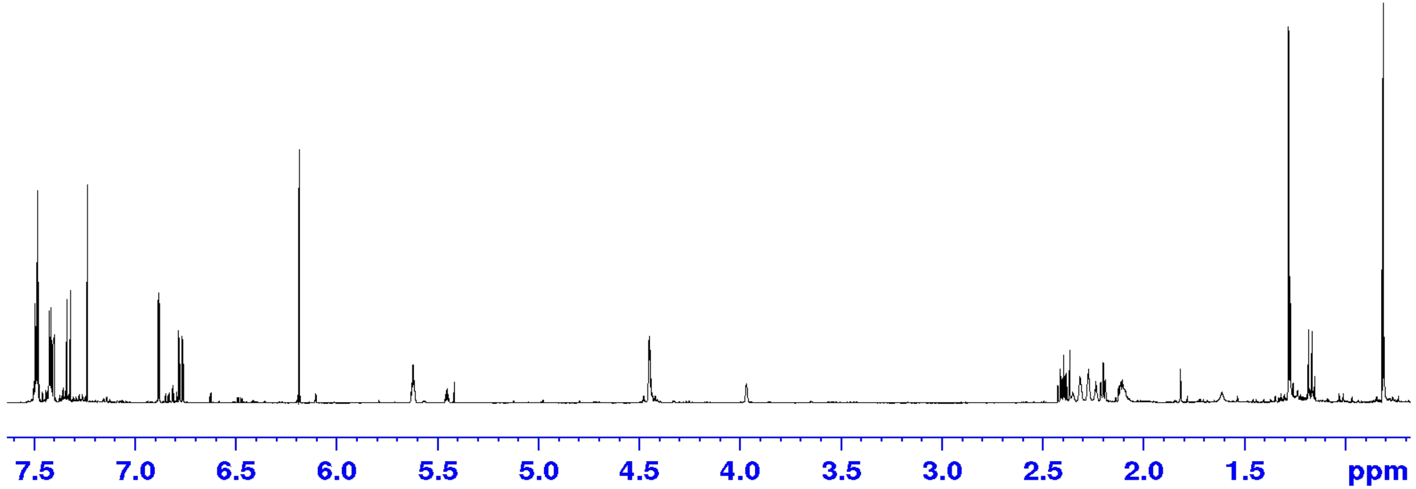

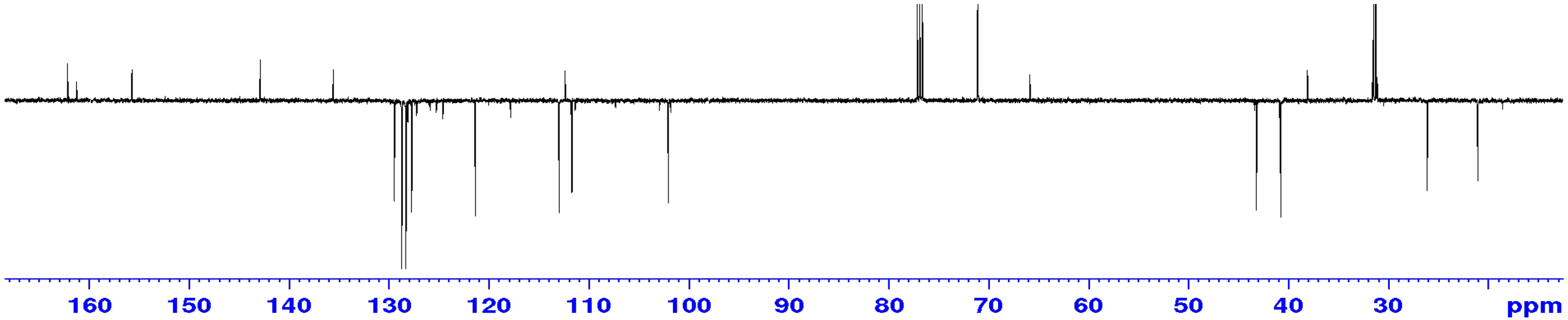

Compound **3bc**

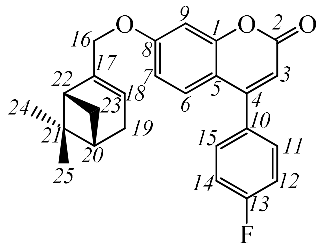

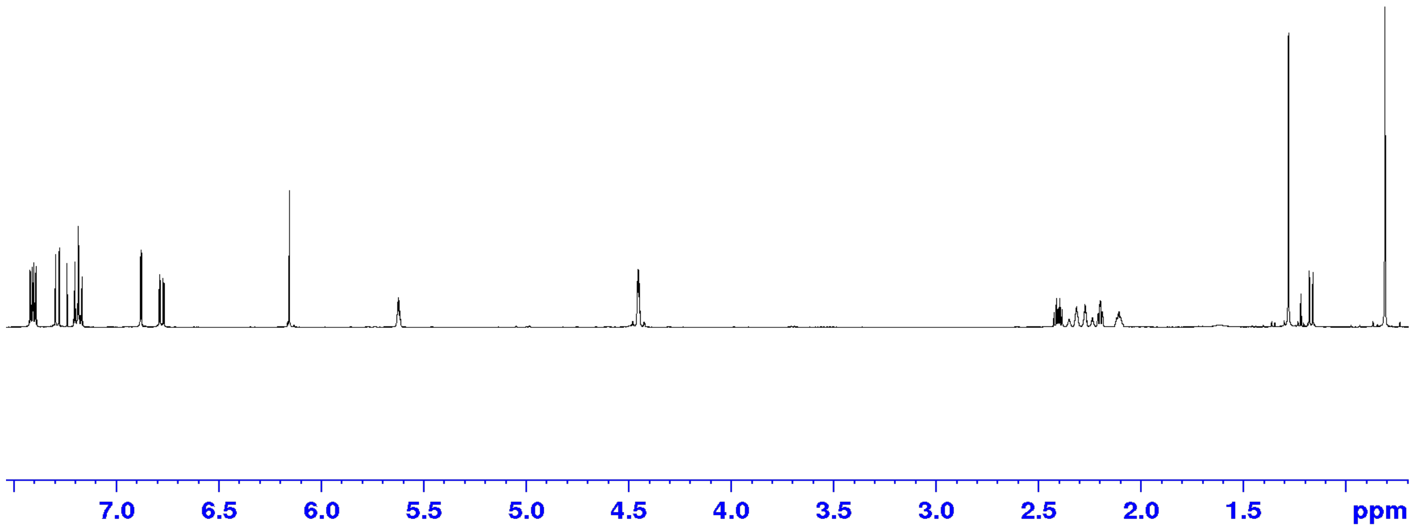

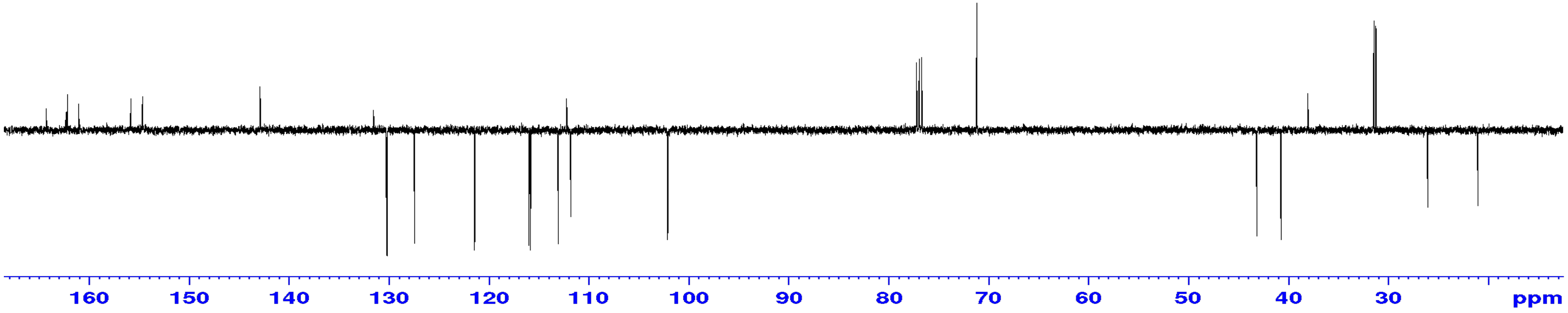

Compound **3cc**

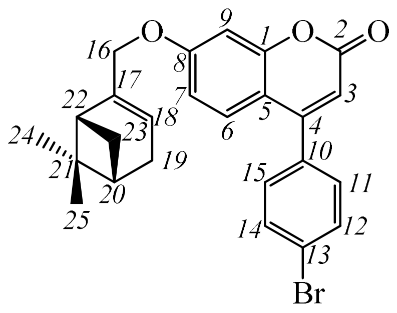

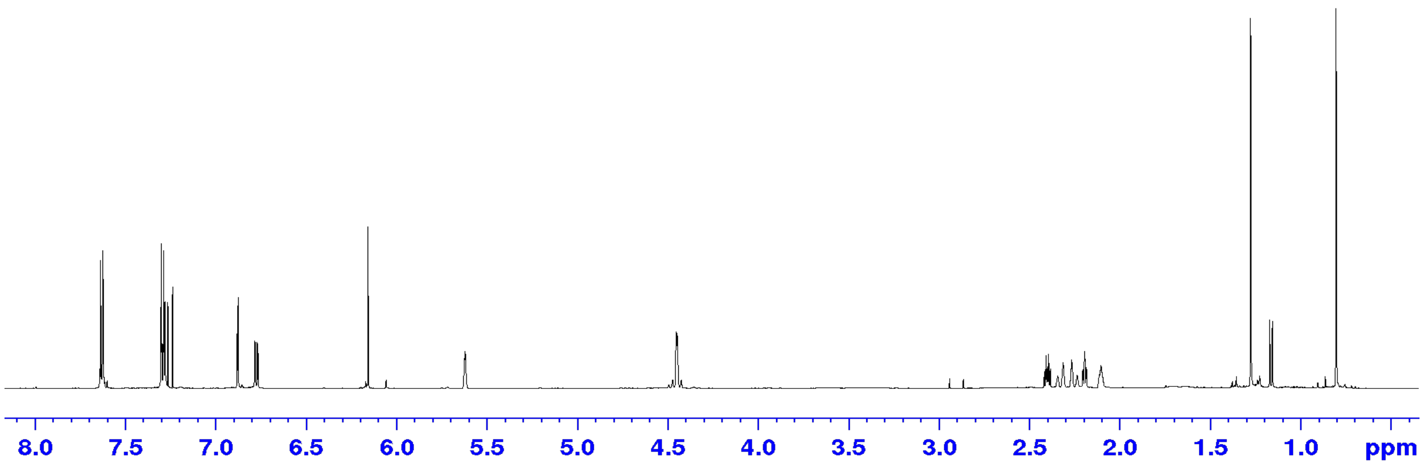

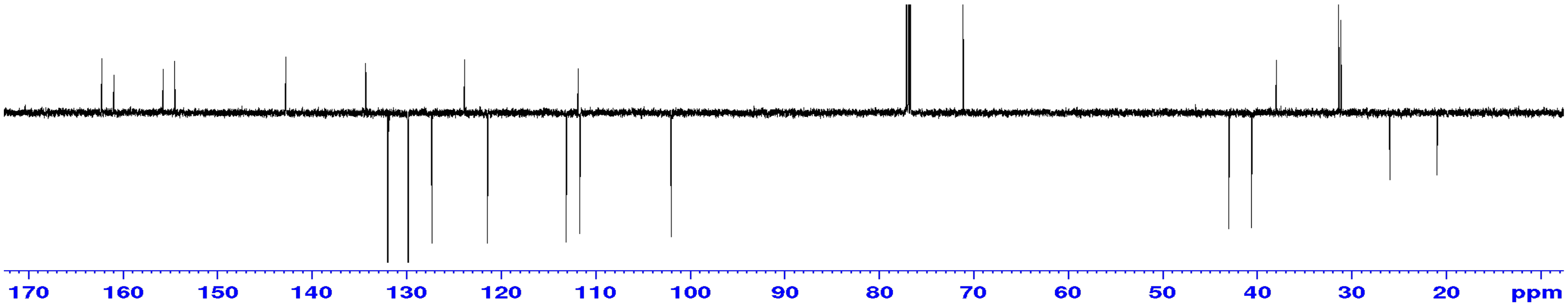

Compound **3dc**

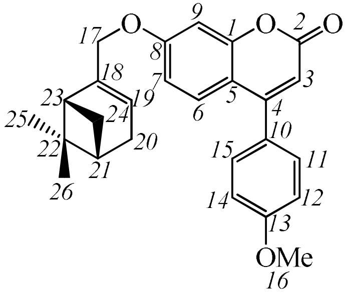

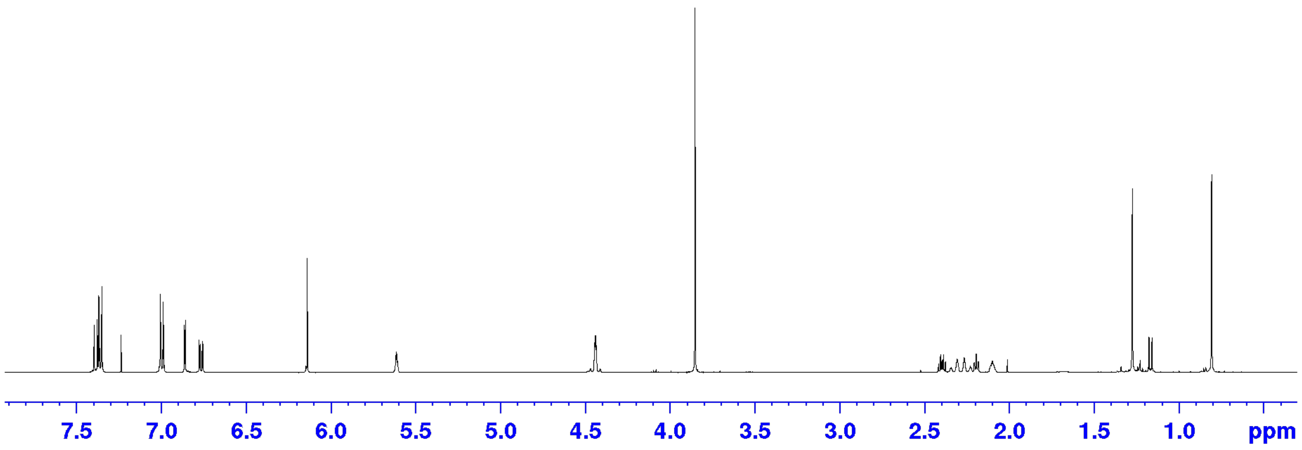

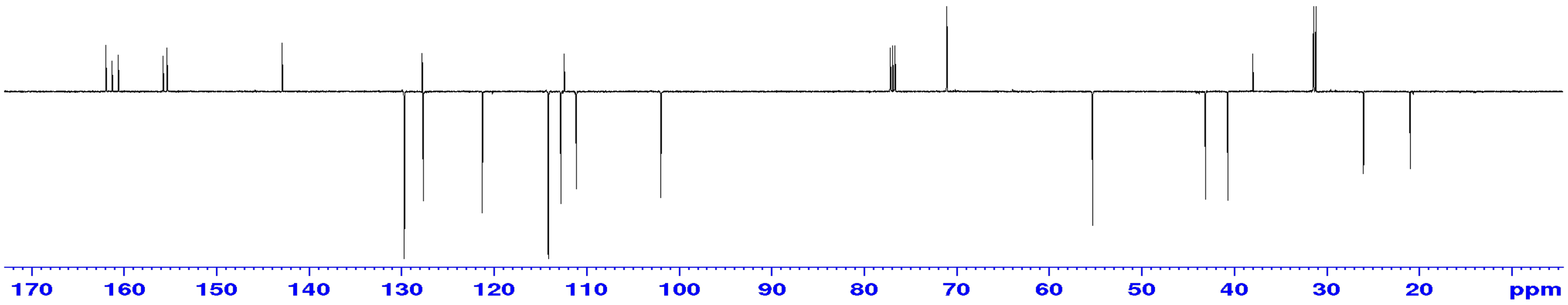

Compound **10a**

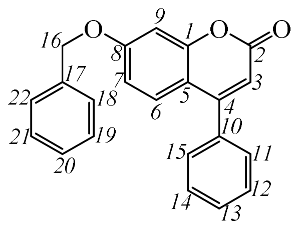

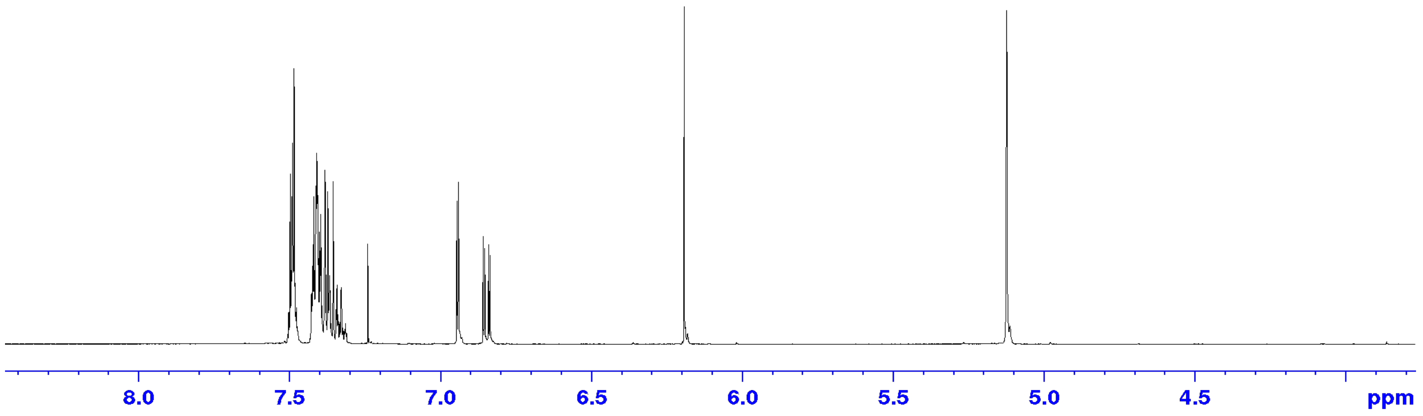

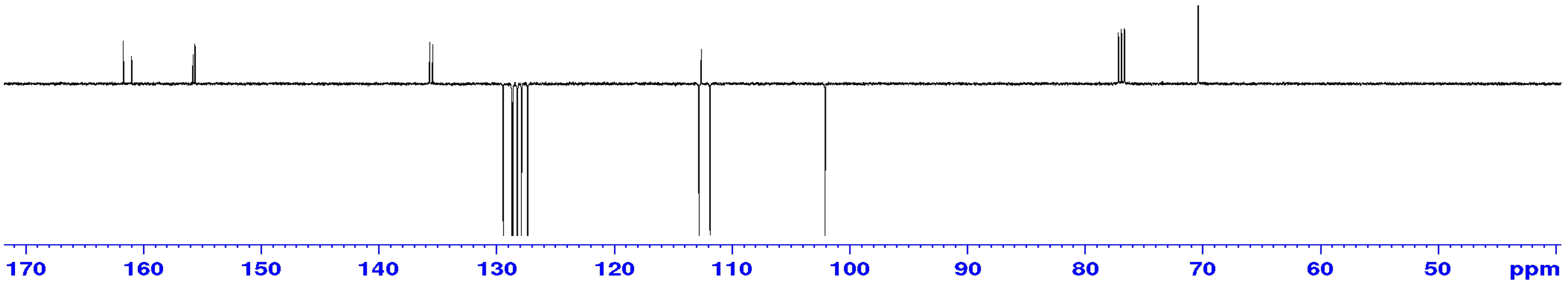

Compound **10c**

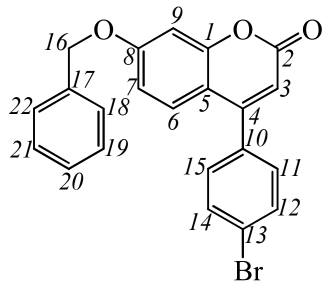

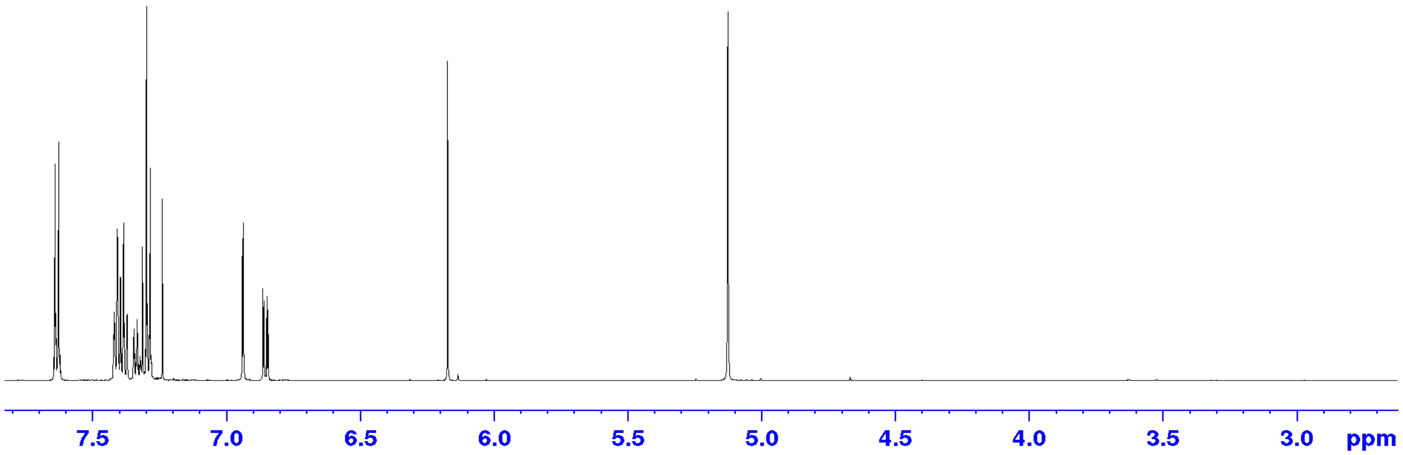

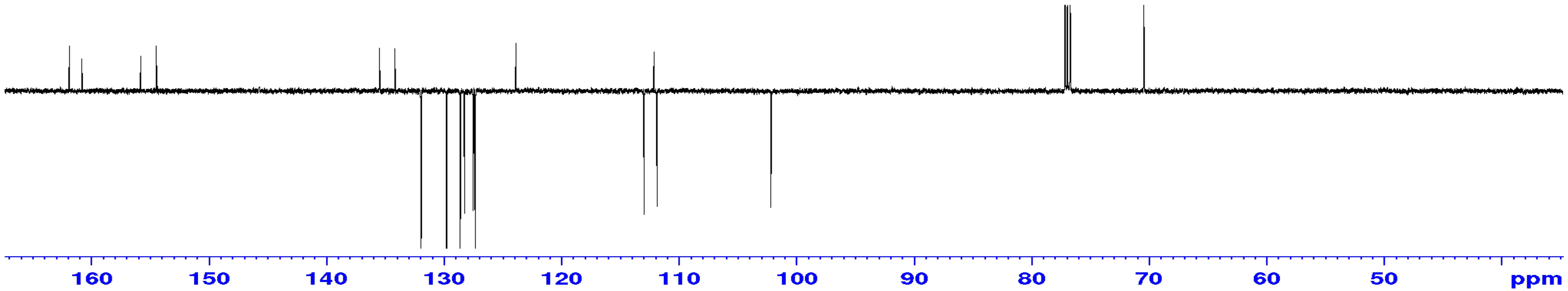

Compound **10d**

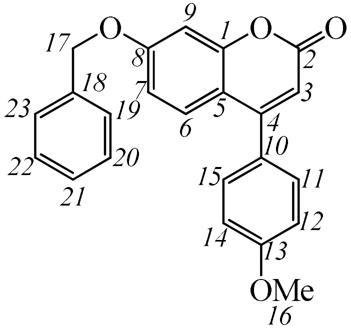

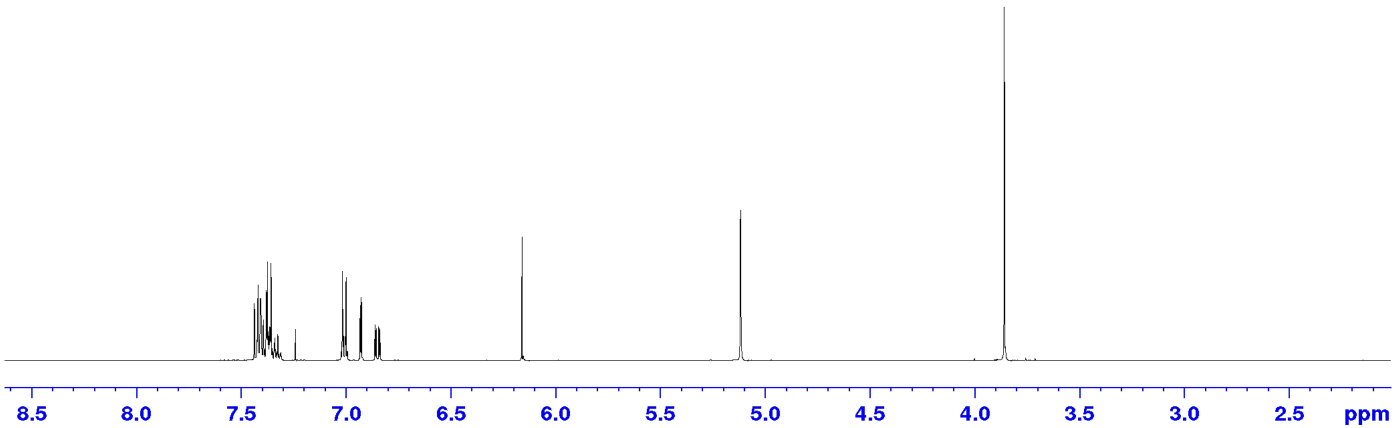

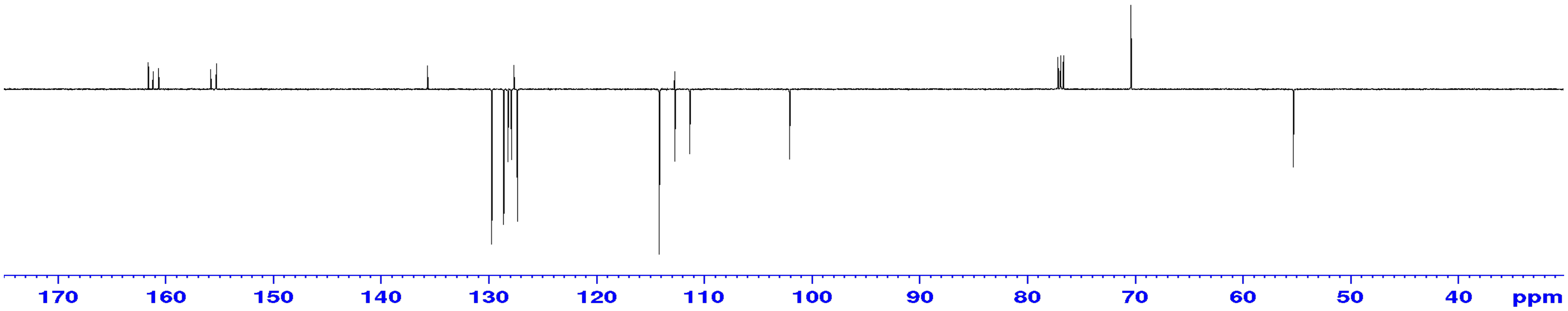

Table 1. The Tdp1 inhibitory activities of compounds **3aa - 3dd** and **10a, c, d**.

Code	IC_{50}, μM
**3aa**	0.49±0.14
**3ba**	0.62±0.06
**3ca**	0.90±0.37
**3da**	0.52±0.05
**3ab**	0.41±0.12
**3bb**	7.50± 0.70
**3cb**	0.59±0.07
**3db**	0.91±0.13
**3ac**	0.46±0.16
**3bc**	0.99±0.30
**3cc**	3.23±0.35
**3dc**	0.59±0.15
**3ad**	0.48±0.11
**3bd**	1.03±0.58
**3cd**	0.43±0.05
**3dd**	0.59±0.06
**10a**	4.29±1.24
**10c**	2.40±0.80
**10d**	3.31± 0.98
**Fur** [1]	1.20 ± 0.30

[1] Furamidine was used as a positive control.

Table 2. The scores for the 19 ligands docked against Tdp1.

	Compound	ASP	CS	GS	PLP
1	**3aa**	39.2	32.5	66.2	70.3
2	**3ba**	39.1	31.6	69.2	75.2
3	**3ca**	36.9	32.1	70.7	71.0
4	**3da**	42.6	33.9	63.9	68.3
5	**3ab**	37.8	32.4	70.0	68.5
6	**3bb**	39.3	32.5	67.8	61.9
7	**3cb**	39.5	34.4	73.3	73.7
8	**3db**	33.4	27.6	68.2	67.0
9	**3ac**	28.7	27.2	64.7	63.7
1[illegible]	**3bc**	38.2	30.8	64.8	67.1
1[illegible]	**3cc**	36.7	31.6	55.6	67.0
1[illegible]	**3dc**	38.7	31.5	64.2	66.9
1[illegible]	**3ad**	36.4	31.2	56.2	68.5
1[illegible]	**3bd**	37.6	30.5	54.1	64.2
1[illegible]	**3cd**	36.7	28.1	57.3	66.1
1[illegible]	**3dd**	38.5	32.0	65.2	64.6
1[illegible]	**10a**	35.7	30.0	66.9	70.7
1[illegible]	**10c**	36.4	25.6	60.8	66.4
1[illegible]	**10d**	38.1	31.4	60.5	68.7

Table 3. The molecular descriptor values for the 19 compounds. .

	Compound	MW	HB DONOR	HB ACCEPTOR	Log P	PSA	Rot. Bonds
1.	**3aa**	374.5	0	3.25	6.0	47.3	7
2.	**3ba**	392.5	0	3.25	6.3	47.3	7
3.	**3ca**	453.4	0	3.25	6.6	47.3	7
4.	**3da**	404.5	0	4	6.1	55.5	8
5.	**3ab**	386.5	0	3.25	5.7	47.3	5
6.	**3bb**	404.5	0	3.25	5.9	47.3	5
7.	**3cb**	416.5	0	4	5.8	55.5	6
8.	**3db**	416.6	0	4	6.1	54.1	6
9.	**3ac**	372.5	0	3.25	5.7	45.5	4
10	**3bc**	390.5	0	3.25	5.6	46.9	4
11	**3cc**	451.4	0	3.25	6.0	46.9	4
12	**3dc**	402.5	0	4	5.5	55.1	5
13	**3ad**	372.5	0	3.25	5.7	45.5	4
14	**3bd**	390.5	0	3.25	5.6	46.9	4
15	**3cd**	451.4	0	3.25	6.0	46.9	4
16	**3dd**	402.5	0	4	5.4	55.1	5
17	**10a**	326.4	0	3.25	4.4	47.3	4
18	**10c**	407.3	0	3.25	5.1	47.2	4
19	**10d**	358.4	0	4	4.6	55.4	5

Table 4. Definition of lead-like, drug-like and Known drug space (KDS) in terms of molecular descriptors. The values given are the maxima for each descriptor for the volumes of chemical space used.

	Lead-like Space	Drug-like Space	Known Drug Space
Molecular weight (g mol^{-1})	300	500	800
Lipophilicity (Log P)	3	5	6.5
Hydrogen bond donors (HD)	3	5	7
Hydrogen bond acceptors (HA)	3	10	15
Polar surface area ($Å^2$) (PSA)	60	140	180
Rotatable bonds (RB)	3	10	17

Table 5. The calculated KDI indexes for the 19 ligands.

	Compound	KDI $_{2A}$	KDI $_{2B}$
1	**3aa**	5.08	0.33
2	**3ba**	4.90	0.27
3	**3ca**	4.72	0.21
4	**3da**	4.94	0.28
5	**3ab**	5.13	0.36
6	**3bb**	5.07	0.33
7	**3cb**	5.16	0.38
8	**3db**	5.08	0.33
9	**3ac**	5.13	0.36
1[illegible]	**3bc**	5.14	0.37
1[illegible]	**3cc**	4.96	0.30
1[illegible]	**3dc**	5.28	0.44
1[illegible]	**3ad**	5.20	0.40
1[illegible]	**3bd**	5.15	0.37
1[illegible]	**3cd**	4.97	0.30
1[illegible]	**3dd**	5.28	0.44
1[illegible]	**10a**	5.32	0.47
1[illegible]	**10c**	5.21	0.41
1[illegible]	**10d**	5.44	0.54

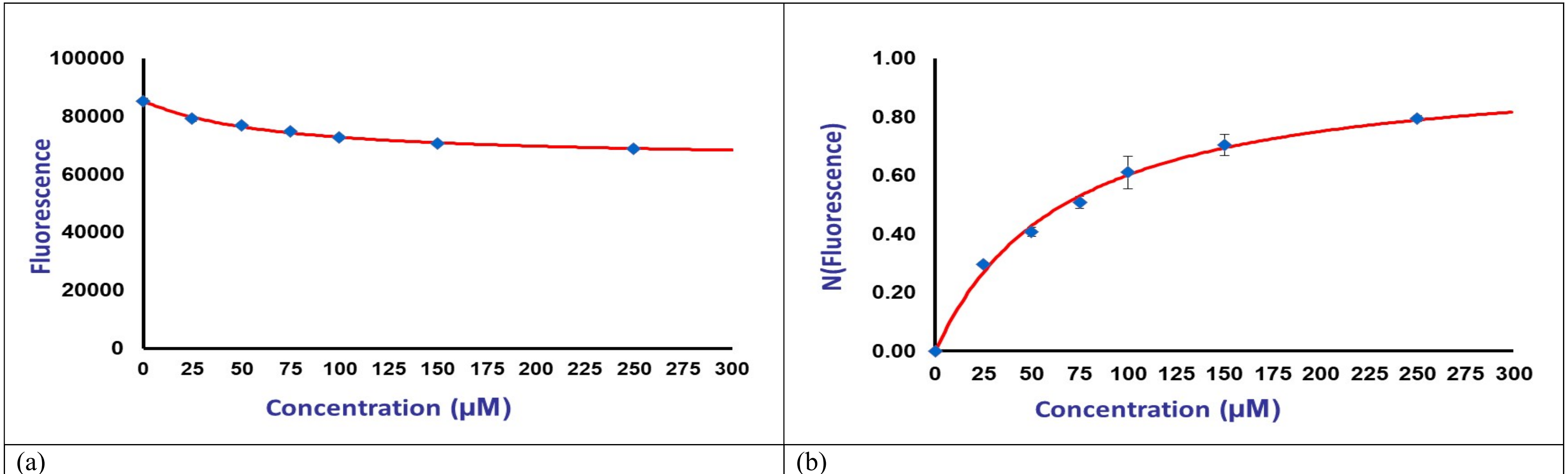

(a) (b)

Supplementary Figure S1. (a) Change in intrinsic fluorescence intensity of Tdp1 (10 µM) upon the addition of compound **3ba** (25 µM, 50 µM, 75 µM, 100 µM, 150 µM and 250 µM). The buffer composition was 20 mM Tris and 250 mM NaCl, pH 8. The excitation wavelength was 280 nm and intrinsic fluorescence was measured at 350 nm. (b) Non-linear curve fitting of the corresponding fluorescence data. The K_D was found to be 63.0 ± 11 µM. Experiment were conducted in triplicate and error shown is standard derivation.
